# Mechanism of Action of Compound-13: An α1-Selective Small Molecule Activator of AMPK

**DOI:** 10.1016/j.chembiol.2014.05.014

**Published:** 2014-07-17

**Authors:** Roger W. Hunter, Marc Foretz, Laurent Bultot, Morgan D. Fullerton, Maria Deak, Fiona A. Ross, Simon A. Hawley, Natalia Shpiro, Benoit Viollet, Denis Barron, Bruce E. Kemp, Gregory R. Steinberg, D. Grahame Hardie, Kei Sakamoto

**Affiliations:** 1 MRC Protein Phosphorylation and Ubiquitylation Unit, College of Life Sciences, University of Dundee, Dow Street, Dundee, DD1 5EH Scotland, UK; 2 Nestlé Institute of Health Sciences SA, EPFL Innovation Park, bâtiment G, 1015 Lausanne, Switzerland; 3 Inserm, U1016, Institut Cochin, 24 rue du Faubourg Saint-Jacques, 75014 Paris, France; 4 CNRS, UMR8104, Paris, France; 5 Université Paris Descartes, Sorbonne Paris cité, 75006 Paris, France; 6 Division of Endocrinology and Metabolism, Department of Medicine, McMaster University, 1280 Main West Street, Hamilton ON L8N 3Z5, Canada; 7 Division of Cell Signalling and Immunology, College of Life Sciences, University of Dundee, Dundee DD1 5EH, UK; 8 Protein Chemistry and Metabolism, St. Vincent’s Institute and Department of Medicine, University of Melbourne, 41 Victoria Parade, Fitzroy VIC 3065, Australia

## Abstract

AMPK is a sensor of cellular energy status and a promising target for drugs aimed at metabolic disorders. We have studied the selectivity and mechanism of a recently described activator, C2, and its cell-permeable prodrug, C13. C2 was a potent allosteric activator of α1-complexes that, like AMP, also protected against Thr172 dephosphorylation. Compared with AMP, C2 caused only partial allosteric activation of α2-complexes and failed to protect them against dephosphorylation. We show that both effects could be fully restored by exchanging part of the linker between the autoinhibitory and C-terminal domains in α2, containing the equivalent region from α1 thought to interact with AMP bound in site 3 of the γ subunit. Consistent with our results in cell-free assays, C13 potently inhibited lipid synthesis in hepatocytes from wild-type and was largely ineffective in AMPK-knockout hepatocytes; its effects were more severely affected by knockout of α1 than of α2, β1, or β2.

## Highlights

• C2 activates AMPK via direct activation and protection against dephosphorylation
• C2 and its cell-permeable prodrug C13 selectively activate AMPKα1
• A sequence located in the C-terminal alpha-linker of α1 confers specificity of C2
• C13 inhibits hepatic lipid synthesis via an AMPK-dependent and α1-selective mechanism

## Teaser

This study reports the selectivity and mechanism of a new AMPK activator. It inhibits hepatic lipid synthesis via an AMPKα1-isoform-selective mechanism and a sequence located in the C-terminal region of α1 that confers specificity.

## Introduction

AMP-activated protein kinase (AMPK) is a central energy sensor and regulator of energy homeostasis (Hardie et al., 2012; Steinberg and Kemp, 2009). AMPK is activated by metabolic stresses that lower cellular energy status by decreasing the catabolic generation of ATP or by accelerating ATP consumption. Upon activation, it functions to restore cellular energy homeostasis by switching off anabolic pathways and other ATP-consuming processes while switching on ATP-producing catabolic pathways.

AMPK is a heterotrimer composed of a catalytic α subunit and regulatory β and γ subunits. Multiple genes encoding isoforms (α1, α2; β1, β2; γ1, γ2, γ3) as well as transcriptional variants exist for each of the subunits, generating at least 12 distinct heterotrimeric complexes (Hardie et al., 2012; Steinberg and Kemp, 2009). There are cell- and tissue-specific expressions of some isoforms, and they may also target AMPK complexes to specific subcellular locations (Hudson et al., 2003; Salt et al., 1998). The γ subunits contain four tandem cystathionine β-synthase (CBS) repeats that provide four potential sites for adenine nucleotide binding, although only three are used (Xiao et al., 2007). AMPK activity increases >100-fold on phosphorylation of a conserved threonine residue within the activation loop (Thr172 in rat α2; Hawley et al., 1996). Binding of ADP and/or AMP causes conformational changes that promote net Thr172 phosphorylation by (1) the promotion of Thr172 phosphorylation and (2) the inhibition of Thr172 dephosphorylation (Gowans et al., 2013; Oakhill et al., 2011; Xiao et al., 2011). In addition, the binding of AMP (but not ADP) further stimulates AMPK activity by >10-fold by allosteric activation (Gowans et al., 2013). The major upstream kinase phosphorylating Thr172 in most mammalian cells is a complex containing the tumor suppressor kinase LKB1, which appears to be constitutively active (Alessi et al., 2006; Sakamoto et al., 2004). In some cells, Thr172 can be phosphorylated in a Ca^2+^-mediated process catalyzed by Ca^2+^/calmodulin-dependent protein kinase kinases (Hardie et al., 2012; Steinberg and Kemp, 2009).

AMPK is considered a major target for drugs to combat the growing epidemic of metabolic disorders (Hardie et al., 2012) because AMPK activation elicits metabolic responses expected to counteract the physiological or metabolic abnormalities associated with obesity, insulin resistance, and type 2 diabetes. For example, AMPK phosphorylates and inactivates acetyl-CoA carboxylase-1 (ACC1) and HMG-CoA reductase, key enzymes of fatty acid and sterol biosynthesis, respectively (Hardie et al., 2012; Steinberg and Kemp, 2009). Moreover, numerous studies have demonstrated that the activation of AMPK leads to increased fatty acid oxidation through phosphorylation of acetyl-CoA carboxylase-2 (ACC2) (Merrill et al., 1997) and glucose uptake in skeletal muscle involving phosphorylation of TBC1D1 (O’Neill et al., 2011; Pehmøller et al., 2009; Sakamoto and Holman, 2008), whereas AMPK signaling to ACC is required for the lipid-lowering and insulin-sensitizing effects of metformin (Fullerton et al., 2013). In line with this, 5-aminoimidazole-4-carboxamide riboside (AICAR), the most widely used pharmacological AMPK activator, which is converted within cells to the AMP-mimetic AICAR monophosphate (ZMP), improved insulin sensitivity in animal models of insulin resistance (Hardie et al., 2012). However, ZMP modulates other AMP-sensitive enzymes of carbohydrate metabolism, including fructose-1,6-bisphosphatase in the liver (Vincent et al., 1991) and glycogen phosphorylase in muscle (Longnus et al., 2003). In fact, some metabolic effects of AICAR have been shown to be AMPK-independent (Foretz et al., 2010; Guigas et al., 2009). Cool et al. (2006) described the identification of the thienopyridone A769662, the first small-molecule direct activator of AMPK. A769662, like AMP, inhibits Thr172 dephosphorylation (Göransson et al., 2007; Sanders et al., 2007). However, this does not appear to be its primary mechanism of AMPK activation because A769662 can allosterically activate AMPK in the absence of Thr172 phosphorylation, either alone or in the presence of AMP, depending on the phosphorylation state of Ser108 on the β1 subunit (Scott et al., 2014a). The strong synergy between AMP and A769662 in allosteric activation of AMPK in cell-free assays (Scott et al., 2014a) is also observed in vivo (Ducommun et al., 2014; Foretz et al., 2010; Timmermans et al., 2014). A769662 binds to a site located between the α subunit kinase domain and the β subunit carbohydrate-binding module, which is distinct from the adenine nucleotide-binding sites on the γ subunit (Xiao et al., 2013). A769662 is rather selective for complexes containing β1 rather than the β2 isoform (Scott et al., 2008). The small-molecule AMPK activator 991 was also shown to bind at the A769662 site (Xiao et al., 2013). We recently reported that salicylate is a direct activator of AMPK and that it binds the same site as A769662 (Hawley et al., 2012). These studies demonstrate that various small molecules can stimulate AMPK activity through different binding sites and mechanisms.

Recently, Gómez-Galeno et al. (2010) screened a library of 1,200 AMP mimetics and identified 5-(5-hydroxyl-isoxazol-3-yl)-furan-2-phosphonic acid (compound 2, C2) as a potent allosteric activator of AMPK. They also showed that a prodrug (compound 13, C13), stimulated AMPK and inhibited hepatic lipogenesis in vivo. However, the study provided little information about the molecular actions of C2 or C13 on AMPK or about its specificity and biological effects in intact cells. We have investigated the effects of C2 on various combinations of recombinant αβγ complexes in cell-free assays and the effects of C13 in primary hepatocytes from wild-type (WT) and AMPK-isoform-specific-knockout mice in order to gain insights into its mechanism of action and overall effects on lipid metabolism. We report that C2 is a potent allosteric activator of α1 complexes that, like the natural activator AMP, also protects against Thr172 dephosphorylation. Unexpectedly, but of significance, C2 is rather selective for α1 complexes in cell-free assays and its cell-permeable prodrug C13 is also a selective activator of α1 complexes in intact cells.

## Results

### Effects of AMP and C2 on Recombinant AMPK Complexes in Cell-Free Assays

**Figure 1:**
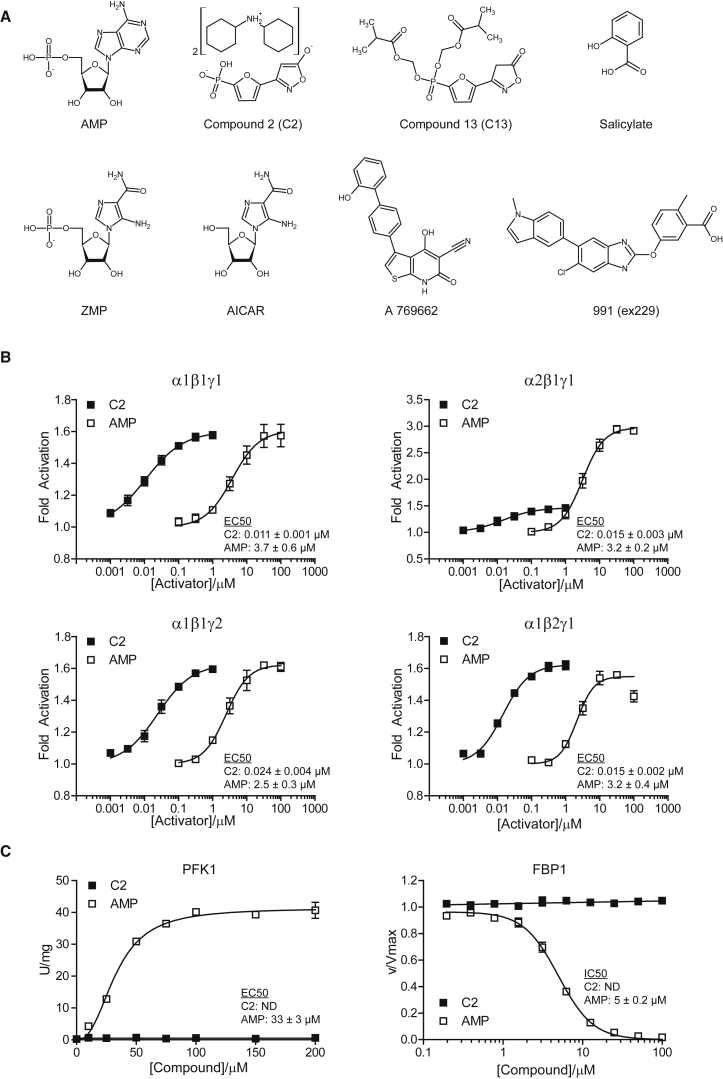
The AMP Analog C2 Is a Potent AMPK Activator in Cell-Free Assays with Selectivity for α1 Complexes (A) The structure of the endogenous activator of AMPK, AMP; the AMP mimetic, C2; and the classical AMP mimetic, ZMP, and other AMPK activators are shown. C2 and ZMP exhibit poor membrane permeability and are administered as the prodrugs, C13 and AICAR, respectively. (B) Recombinant AMPK complexes (α1β1γ1, **α2**β1γ1, α1β1**γ2**, and α1**β2**γ1) expressed in *Spodoptera frugiperda* were assayed for allosteric activation by AMP or C2. Results are expressed as the increase in activity relative to controls without ligand and represent the mean ± SD for three independent experiments. (C) Enzymes allosterically regulated by AMP (PFK1 and FBP1) or using AMP as a substrate (AMPD1, AK, and 5′-NT) were assayed in the presence of C2 (≤ 100 μM). Enzymes allosterically regulated by AMP were screened for both agonism and antagonism, the latter in the presence of AMP at ≈ EC_50_. All enzymes were unaffected by [C2] ≤ 100 μM. Representative data are shown for the effects of AMP and C2 on PFK1 and FBP1 activity. Results are representative of three independent experiments conducted on one enzyme preparation.

The structure of the endogenous activator of AMPK, AMP, is shown in Figure 1A along with C2, 991, salicylate, and the classical AMPK tool compound, ZMP, which is generated by phosphorylation by cellular enzymes of the prodrug, AICAR. C2 bears close structural similarity to AMP, with an acidic 5-hydroxyisoxazole group in place of adenine. It is structurally distinct from the prototypical non-nucleotide AMPK activator, A769662 (Figure 1A). However, in common with ZMP, the charged nature of C2 results in poor membrane permeability, and it is administered in cell-based and in vivo analyses in the form of an esterase-sensitive phosphonate prodrug, C13 (Figure 1A). C2 was reported to activate human AMPK with half-maximal effective concentration (EC_50_) at 6.3 nM, but the exact isoform combination used and whether it was purified from bacteria, insect, or mammalian cells were not specified (Gómez-Galeno et al., 2010).

We initially compared the ability of C2 and AMP to activate various recombinant human AMPK complexes, expressed in insect cells, in cell-free assays (Figure 1B). As reported previously using rat liver complexes separated by immunoprecipitation (Salt et al., 1998), the allosteric activation of α1 complexes by AMP was less than that of α2 complexes. Despite this difference, C2 and AMP were equally effective in allosteric activation of the major α1-containing complexes (α1β1γ1, α1β2γ1, and α1β1γ2), although C2 was two orders of magnitude more potent than AMP, with an EC_50_ of 10–30 nM, compared to 2–4 μM for AMP. Unexpectedly, C2 was only a partial agonist of α2-containing complexes compared with AMP, exhibiting only 15% of the maximal response to saturating AMP using the α2β1γ1 complex. Similar results were obtained with other permutations of β and γ subunits in complex with α2 (data not shown). We also tested the effects of C2 and AMP on recombinant human α1β1γ1 and α2β1γ1 complexes expressed in *E. coli* and obtained similar results (data not shown). The small, partial activation of the α2β1γ1 complex was also potent (EC_50_ of 15 nM, compared to 3 μM for AMP). Characterization of the subunit composition of the recombinant complexes used in this study is shown in Figure S1(available online).

### Effects of C2 on Other AMP-Regulated Enzymes and Protein Kinases

AMP (and ZMP) are known to allosterically modulate several enzymes other than AMPK; for example, AMP activates 6-phosphofructo-1 kinase (PFK1), and AMP and ZMP inhibit the gluconeogenic enzyme fructose-1,6-bisphosphatase-1 (FBP1) (Vincent et al., 1991). As expected, AMP activated PFK1 with an EC_50_ of 33 μM and inhibited FBP1 with a half-maximal inhibitory concentration (IC_50_) of 5 μM (Figure 1C). By contrast, C2 had no effect on PFK1 and FBP1 at concentrations up to 100 μM (Figure 1C), nor did it antagonize the effects of AMP on these enzymes (data not shown). Moreover, we have tested the effect of C2 on another AMP-regulated enzyme (muscle glycogen phosphorylase b) and enzymes using AMP as substrate (AMP deaminase-1, adenylate kinase, and 5′-nucleotidase). None of these enzymes were affected by C2 at concentrations up to 100 μM (data not shown).

To determine whether C2 affects the activity of any other protein kinases, we screened it in cell-free assays against a panel of 138 protein kinases. The majority were not affected by 10 μM C2 (Figure S2), including several that are members of the AMPK-related kinase family (SIK2, SIK3, NUAK1, MELK, MARK1, MARK2, MARK3, MARK4, BRSK1, and BRSK2). Moreover, C2 did not affect any of the known upstream kinases of AMPK, including LKB1, CaMKKβ, and TAK1 (Figure S2). A few kinases were marginally inhibited by C2 at 10 μM, which is ≈10-fold higher than the concentration that is saturating for AMPK activation in cell-free assays (Figure 1B). Taken together, these results suggest that C2 is a rather specific AMPK activator.

### C2 Is a Partial Agonist of AMPKα2 Complexes and Does Not Protect Their Activation Loops from Dephosphorylation

**Figure 2:**
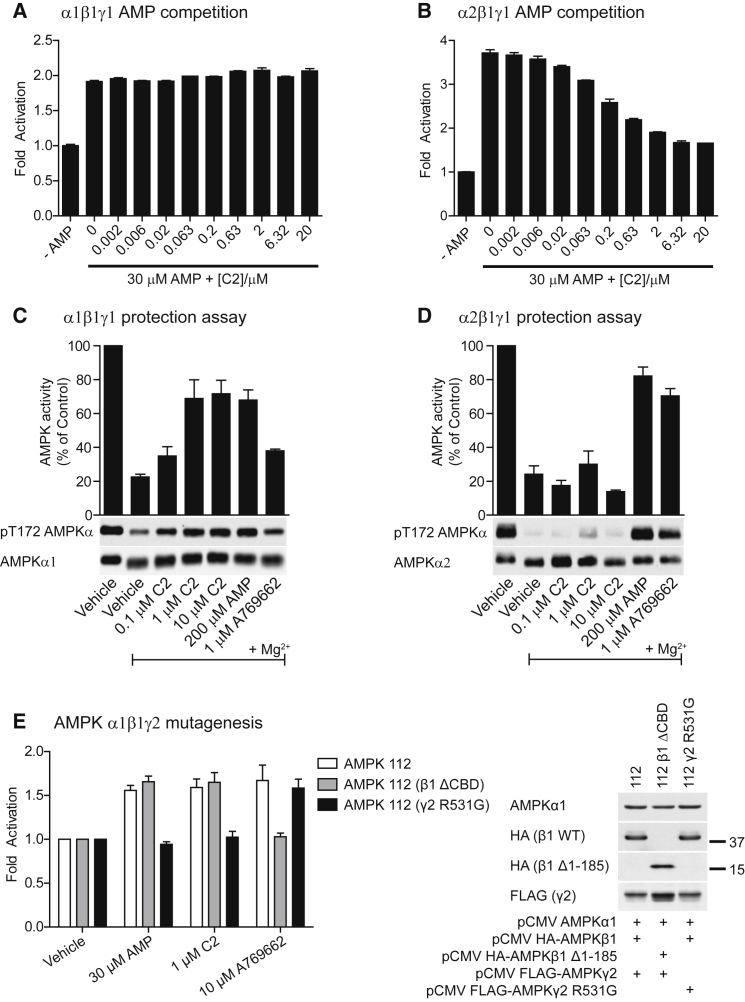
C2 Is a Partial Agonist of α2 Complexes and Selectively Protects α1 Complexes against Dephosphorylation by PP2C (A and B) Recombinant AMPKα1β1γ1 (A) or AMPKα2β1γ1 (B) was assayed in the presence of AMP (30 μM) and increasing concentrations of C2 (0–20 μM). Results are expressed as fold increase in activity relative to controls without ligand and represent the mean ± SD for three independent experiments. (C and D) The effects of C2 and AMP on dephosphorylation and inactivation of AMPKα1β1γ1 (C) and α2β1γ1 (D) by PP2Cα (present in all assay conditions). Results are expressed as a percentage of control reactions performed in the absence of Mg^2+^ and represent the mean ± SD of three independent experiments. Representative blots of pT172 and total AMPKα are shown below the bar charts. (E) AMPKα1β1γ2 WT (open bars) and mutant complexes (β1 ΔCBD [β1 Δ1–185], gray bars, and γ2 R531G, black bars) were purified from COS1 cells by transient overexpression of the indicated constructs, as described in Methods, and assayed in the presence of the indicated compounds under standard conditions. Results are expressed as the fold increase in activity relative to controls in the absence of compound and represent the mean ± SD for three independent experiments. The right-hand panel shows an analysis of the subunit composition of the preparations by western blotting with the indicated antibodies.

Speculating that C2 exerts its effects by exploiting the same binding site on the γ subunits as AMP, we found the modest allosteric activation of α2 complexes by C2 compared to AMP unexpected. One explanation is the presence of a second, inhibitory site unique to α2. Indeed, at the low concentrations of ATP typically used in radiometric kinase assays, activation by AMP itself is biphasic, with a pronounced inhibitory effect at high concentrations due to competition with ATP at the catalytic site (Gowans et al., 2013). However, C2 had no effect on the activity of isolated, full-length α subunits when assayed under identical conditions to Figure 1B (Figure S3A), showing that it does not compete with ATP at the catalytic site. Gómez-Galeno et al. (2010) reported that C2 activated only partially an AMPK preparation from rat liver. Similarly, we found that C2 activated rat liver AMPK to only half the extent that AMP did (Figure S3B), which is probably because this preparation contains a roughly equal mixture of α1β1γ1 and α2β1γ1 complexes (Woods et al., 1996). In addition, C2 antagonized activation by AMP, reducing the activity of rat liver AMPK stimulated by 30 μM AMP by ≈50%, as expected for a partial agonist (Figure S3C). This was confirmed using isolated α1β1γ1 and α2β1γ1 complexes (Figures 2A and 2B); increasing concentrations of C2 had no effect on the activity of an α1β1γ1 complex measured in the presence of 30 μM AMP, but it reduced the activity of an α2β1γ1 complex to ≈15% above basal (similar to the maximum effect of C2 alone on this complex; Figure 1B). These results support the assumption that C2 and AMP share at least one mutual binding site or transduction mechanism. Consistent with this, activation of α1β1γ1 by C2 was antagonized by ADP (Figure S3D), which also binds to the γ subunit but does not elicit an allosteric response and behaves as a competitive antagonist under these conditions (Xiao et al., 2011). We also observed that C2 failed to stimulate an AMPK complex containing a point mutation in the γ2 subunit (R531G), which renders AMPK complexes insensitive to AMP (Sanders et al., 2007), whereas activation by C2 was unaffected on AMPK containing a carbohydrate-binding-domain (CBD)-deletion mutant in the β1 subunit (β1 Δ1–185, which renders AMPK complexes insensitive to A769662; Sanders et al., 2007; Scott et al., 2008) (Figure 2E). Moreover, C2 and AMP displaced a GST-AMPKγ2 subunit fusion from ATP-γ-Sepharose to the same extent, indicating that both ligands compete for the same site(s) as ATP on the isolated γ2 subunit (Figure S3E).

In addition to allosterically activating AMPK (a key part of the overall activation mechanism; Gowans et al., 2013), AMP binding also promotes an increase in Thr172 phosphorylation, mainly by protecting the complex against dephosphorylation by protein phosphatases. To test whether binding of C2, like AMP, inhibited the dephosphorylation of Thr172, the α1β1γ1 complex was incubated with protein phosphatase PP2Cα in the presence or absence of AMP or C2. As expected, AMP provided partial protection against Thr172 dephosphorylation, and we now show that C2 also afforded partial protection at 1 μM (Figure 2C). Interestingly, C2 had essentially no effect on the dephosphorylation of an α2β1γ1 complex (Figure 2D) at concentrations (1–10 μM) where the previously observed modest allosteric activation (see Figure 1B) was maximal. As expected, C2 was only partially effective when similar assays were performed using the rat liver preparation (data not shown). We also verified that these concentrations of C2 had no direct effect on PP2Cα activity, assayed using a synthetic peptide substrate corresponding to the T loop sequence of AMPKα1/α2. The plant alkaloid sanguinarine was recently reported to be a PP2C inhibitor (Aburai et al., 2010) and was included as a positive control (Figure S3F).

### C2 Can Be Rendered a Full Agonist of α2 Complexes by Substitution of Regulatory Elements from α1

**Figure 3:**
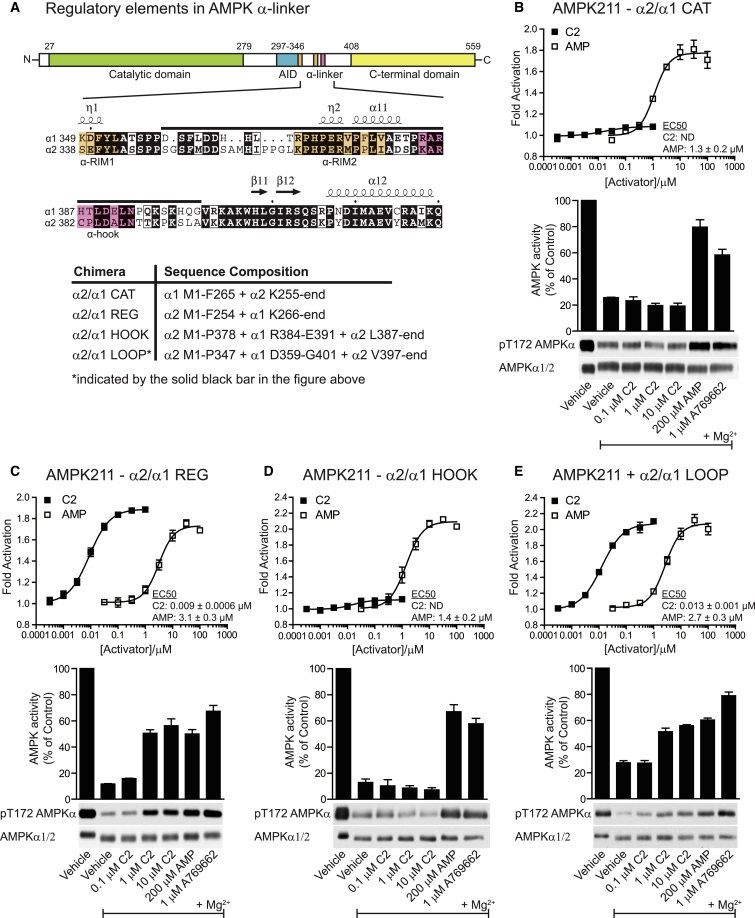
Regulatory Elements within the α-Linker Determine the Isoform Specificity of C2 (A) Diagram illustrating the domain organization of the AMPKα subunit with a global pairwise alignment of a section of the α-linker of human AMPKα1 (Q13131) and AMPKα2 (P54646). Regulatory elements in the α-linker are highlighted, including the α-hook as originally defined by Xiao et al. (2011) (pink) and the reassigned sequence (also known as α-RIM2) shaded in orange. The secondary structure for α1 is derived from PDB ID 2Y94. The table summarizes the sequences of the α1/α2 chimeras produced to examine the role of various structural elements in the activation of α2β1γ1 complexes by C2. (B–E) Human AMPKα1/α2 chimeras (defined in A) were generated as complexes with β1γ1 in *E. coli*. Purified active complexes were assayed for the activation of phosphotransferase activity by AMP and C2, as described in Figure 1. Results (line graphs) are expressed as the fold increase in activity relative to reactions performed in the absence of compound and represent the mean ± SD of three independent experiments. Complexes were also assayed for protection against dephosphorylation and inactivation by PP2Cα, as described in Figures 2C and 2D. Results (bar charts) are expressed as the mean (percentage of control) ± SD of three independent experiments. Representative blots of pT172 and total AMPKα are shown below the bar charts.

The data shown in Figure 2E and Figure S3E suggest that C2 functions through binding to the γ subunit and that the poor response of α2 versus α1 complexes to the compound (relative to AMP) may be due to the different sequences of α1 and α2 isoforms in the region that contacts the AMP-binding domains of the γ subunit. Structures of active heterotrimeric complexes containing α1 or α2 (Xiao et al., 2011, 2013) revealed that the α linker, which connects the autoinhibitory and C-terminal domains (α-AID and CTD) of the α subunit, wraps around one face of the γ subunit, contacting AMP bound in site 3. In the original model (Xiao et al., 2011), a region termed the α hook (α1 384–393, Figure 3A and Figure S4) was proposed to contact AMP bound in site 3 (Xiao et al., 2011). While our study was in progress, this model was revised and the sequence corresponding to the α hook was reassigned (also referred to as α-RIM2) (Chen et al., 2013; Xiao et al., 2013), as discussed in more detail in the Discussion section. However, the original model was used to guide the experiments described in the next paragraph.

To identify the region that determines the isoform specificity of C2, we designed and prepared a series of recombinant complexes with chimeric α subunits comprising combinations of the catalytic and regulatory elements (Figure 3A; Figure S4). The substitution of α2β1γ1 with the catalytic domain from α1 (α2/α1 CAT) had no significant impact on the ability of C2 to allosterically activate AMPK (Figure 3B, top) or protect against dephosphorylation by PP2Cα (Figure 3B, bottom), confirming that the poor activation of α2 complexes by C2 is not because it antagonizes ATP binding at the catalytic site. However, when the region of α2 C-terminal to the kinase domain was replaced with the complementary region from α1 (α2/α1 REG), the complex was fully activated by C2 with an EC_50_ comparable to α1β1γ1 (Figure 3C, top) and was as effective as AMP at protecting against dephosphorylation (Figure 3C, bottom). When we limited the substitution of the C-terminal region of α2 to the α hook of α1 (α2/α1 HOOK), the rescue was lost, with no discernible impact on either allosteric activation or protection against dephosphorylation (Figure 3D). However, a full rescue could be realized by substituting the full region of low similarity between α1 and α2, (α2/α1 LOOP, which includes both the α-RIM2 and α hook sequences) (Figure 3E). This yielded results essentially identical to substituting the entire C-terminal region (Figure 3C). These results were confirmed in intact cells by the transient overexpression of FLAG-α2, β1, and γ1 in COS1 cells treated with the C2 prodrug, C13 (Figure S4B). Cells expressing WT FLAG-α2 were unresponsive up to 100 μM C13, whereas FLAG-α2/α1 LOOP complexes were activated by C13 treatment to a similar extent as AICAR (used in combination with methotrexate to increase ZMP accumulation). These results highlight a hitherto unrevealed difference between the regulatory apparatus of α1 and α2 complexes, and indicate the potential for exploiting these differences to design isoform-selective AMPK activators.

### Effects of the C2 Prodrug (C13) on AMPK Signaling in Primary Mouse Hepatocytes

Previous studies have shown that C2 displayed no significant cellular accumulation in primary hepatocytes when used at 100 μM for up to 6 hr (Gómez-Galeno et al., 2010), suggesting that it has poor cell permeability, perhaps due to its anionic nature. Gómez-Galeno et al. (2010) synthesized a series of prodrugs in which the phosphate moiety was derivatized using esterase-sensitive groups. Among these, C13 (Figure 1A) displayed the most potent inhibition of whole-body lipogenesis in mice, and thus we chose it for our cell-based studies.

**Figure 4:**
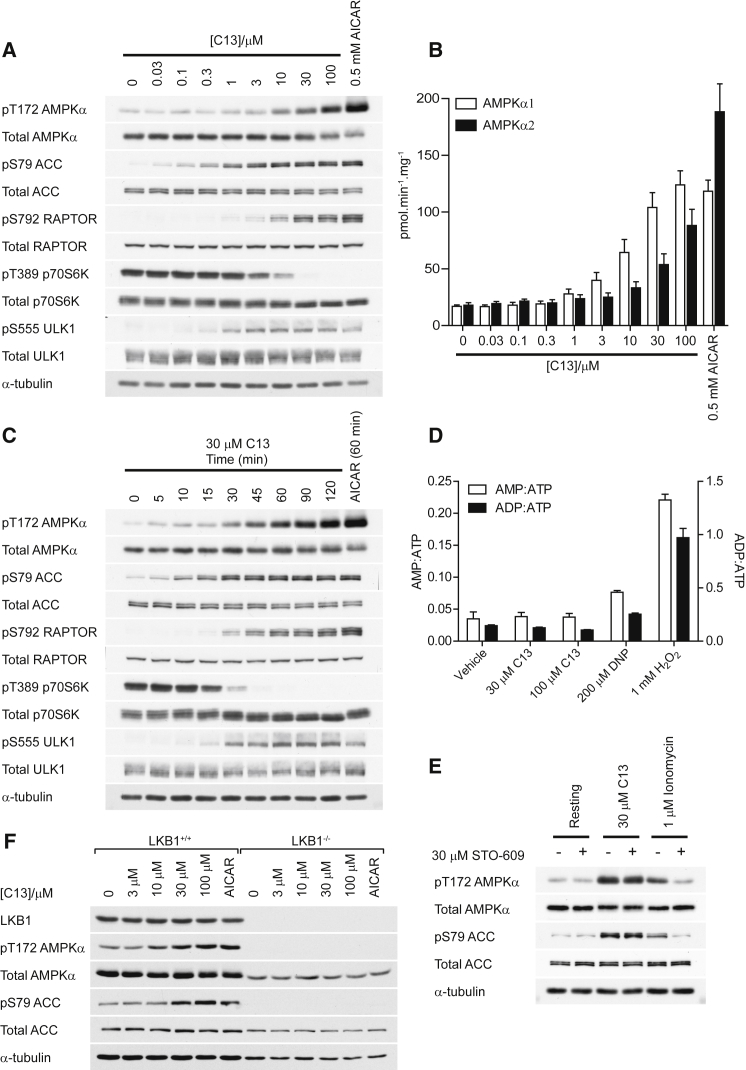
The C2 Prodrug, C13, Potently Activates AMPK in Mouse Primary Hepatocytes (A) Isolated mouse hepatocytes were incubated with vehicle or the indicated concentrations of C13 for 1 hr. AICAR (0.5 mM) was included as a positive control. Cell lysates were analyzed using western blotting with the indicated antibodies. (B) AMPKα1 or AMPKα2 complexes were immunoprecipitated from hepatocyte extracts prepared as described in (A) and assayed for kinase activity using 0.2 mM AMARA and 0.1 mM ATP. Results are expressed as the mean P_*i*_ incorporated in picomoles per minute-milligram ± SD. (C) Hepatocytes were stimulated with 30 μM C13 for the indicated times prior to harvesting, with AICAR (0.5 mM, 1 hr) as positive control. Lysates were analyzed using western blotting with the indicated antibodies. (D) Mouse hepatocytes were treated with the indicated compounds for 1 hr, and adenine nucleotide ratios were determined using capillary electrophoresis of perchloric acid extracts. (E) Mouse hepatocytes were preincubated with 30 μM STO-609 for 30 min prior to stimulation with 30 μM C13 for 1 hr or with 1 μM ionomycin for 30 min. Lysates were analyzed using western blotting with the indicated antibodies. (F) Wild-type (LKB1^+/+^) or LKB1-null (LKB1^−/−^) mouse embryonic fibroblasts were incubated with the indicated concentrations of C13 for 1 hr. AICAR (2 mM) was included as a positive control. Lysates were analyzed using western blotting with the indicated antibodies. Results are representative of three independent experiments.

When mouse primary hepatocytes were incubated with various concentrations of C13 for 1 hr, we observed a modest elevation of Thr172 phosphorylation at concentrations as low as 10 μM and a concentration-dependent increase up to 100 μM (Figure 4A). Thr172 phosphorylation at 100 μM was lower than with 0.5 mM AICAR. Phosphorylation of ACC, a marker for AMPK activation, was evident at concentrations above 0.03–0.1 μM and appeared to be saturated at 1–3 μM. By contrast, other AMPK substrates (i.e., Raptor and ULK1) were significantly phosphorylated only at concentrations above 1–3 μM. Because AMPK is thought to inhibit the mammalian target of rapamycin complex 1 (mTORC1) pathway via the phosphorylation of Raptor and TSC2 (Hardie et al., 2012), we also assessed the phosphorylation of Thr389 on p70S6K1, a marker for mTORC1 activation. We observed that Thr389 phosphorylation was suppressed at concentrations >1 μM, correlating inversely with Raptor phosphorylation (Figure 4A). We also found that C13 suppressed insulin-stimulated mTORC1 activation, as judged by phosphorylation of p70S6K and 4EBP1 (Figure S5).

Given that C2 more effectively activated recombinant α1 than α2 complexes in cell-free assays, we wished to examine whether it would also preferentially activate α1 complexes in intact cells. Indeed, C13 stimulated α1 complexes in primary hepatocytes at much lower concentrations (3 μM) than α2 complexes, whose activation was evident only at concentrations above 30 μM (Figure 4B). Note that in these assays, which were conducted in washed immunoprecipitates made using isoform-specific antibodies, any allosteric activation by C2 or by endogenous AMP would be lost, so the activity is a reflection only of increased Thr172 phosphorylation. A time course at saturating C13 (30 μM) revealed that Thr172 phosphorylation continually increased up to 2 hr, whereas the phosphorylation of downstream targets (ACC, Raptor, and ULK1) was maximal within 45–60 min (Figure 4C).

We next sought to explore the mode of action of C13 in stimulating AMPK. We first assessed ADP:ATP and AMP:ATP ratios. As expected, 2,4-dinitrophenol (DNP) and H_2_O_2_ increased these ratios (Figure 4D), but there were no detectable changes when hepatocytes were incubated with C13 at concentrations up to 100 μM for 1 hr. Activation of AMPK requires Thr172 phosphorylation, and this is primarily mediated by LKB1 or CaMKKβ. We first examined the requirement for CaMKKβ using the relatively selective CaMKK inhibitor, STO-609. Ionomycin, which activates AMPK via increased intracellular [Ca^2+^] and activation of CaMKKβ, was used as a positive control. Prior incubation of hepatocytes with STO-609 almost completely abolished the phosphorylation of AMPK and ACC by ionomycin, but not C13 (Figure 4E). We next measured C13-stimulated AMPK phosphorylation in WT and LKB1^−/−^ mouse embryonic fibroblasts. Both AICAR- and C13-induced phosphorylation of AMPK and ACC were abolished in LKB1^−/−^ cells (Figure 4F).

### C13 Inhibits Lipogenesis and Fatty Acid Esterification

**Figure 5:**
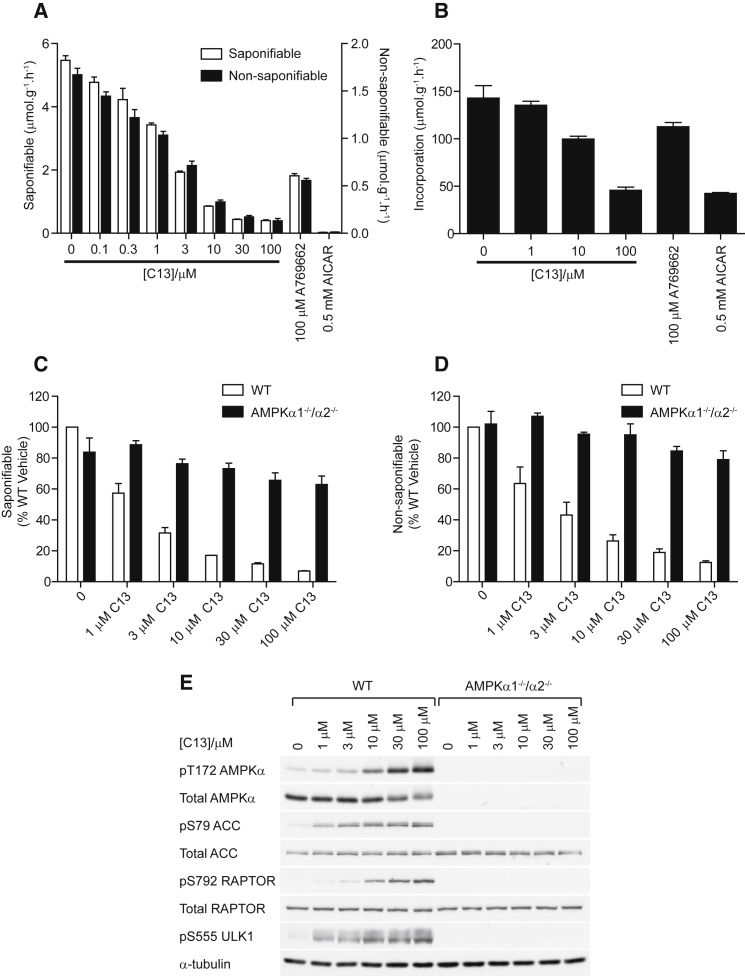
C13 Inhibits Lipid Synthesis and Fatty Acid Esterification in Mouse Hepatocytes in an AMPK-Dependent Manner (A) Mouse hepatocytes were treated with the indicated concentrations of C13 and labeled with [1-^14^C]acetate for 3 hr. A769662 (100 μM) and AICAR (0.5 mM) were included as positive controls. Rates of fatty acid and sterol synthesis were estimated by incorporation into saponifiable and nonsaponifiable lipids. Results are expressed as the mean acetate incorporated in micromoles per gram-hour ± SD and are representative of two independent experiments. (B) Mouse hepatocytes were treated with the indicated compounds for 30 min and labeled with media containing 0.5 mM palmitic acid (1 mCi⋅mmol^−1^ [9,10-^3^H]palmitic acid) for an additional 60 min. Incorporation into triglyceride was determined as described in Experimental Procedures and results are expressed as palmitate incorporated in micromoles per gram-hour ± SD and are representative of three independent experiments. (C and D) Mouse hepatocytes from WT or AMPK-null (AMPKα1^−/−^ -α2^−/−^) mice were incubated with the indicated concentrations of C13 and labeled with [1-^14^C]acetate for 3 hr. Rates of fatty acid and sterol synthesis were estimated from incorporation into saponifiable (C) and nonsaponifiable (D) lipids. Results are expressed as percentage WT vehicle and represent the mean ± SD for three independent experiments. (E) Hepatocytes were treated with the indicated concentrations of C13 for 3 hr, and lysates were blotted with the indicated antibodies.

One of the best-characterized physiological consequences of AMPK activation is the suppression of hepatic fatty acid and sterol synthesis by phosphorylation of the classical substrates, ACC and HMG-CoA reductase. Primary mouse hepatocytes were incubated with C13, and [^14^C]acetate incorporation into saponifiable lipid (fatty acids) and nonsaponifiable lipids (principally sterols) was assessed. There was a concentration-dependent inhibition of lipid synthesis in response to C13 (IC_50_ of 1.7 μM for saponifiable lipids and 1.5 μM for nonsaponifiable lipids), with a maximal effect at 30 μM (Figure 5A). Based on a similar degree of inhibition with 3 μM C13 and 100 μM A769662 (Figure 5A), C13 appeared to be ≈30-fold more potent than A769662. We also measured the effect of C13 on fatty acid esterification by assessing [^3^H]palmitic acid incorporation into triglycerides. C13 inhibited fatty acid esterification (Figure 5B), as previously observed in rat hepatocytes using AICAR (Muoio et al., 1999).

### AMPK Is Required for Inhibition of Lipid Synthesis by C13

To confirm that inhibition of lipid synthesis by C13 is mediated by AMPK, we isolated primary hepatocytes from liver-specific AMPKα1^−/−^ -α2^−/−^ (AMPK-knockout, AMPK-KO) mice or WT controls. C13 dose-dependently inhibited the synthesis of saponifiable and nonsaponifiable lipids in WT hepatocytes, correlating with increases in the phosphorylation of AMPK and ACC (Figures 5C–5E). Conversely, AMPK-KO hepatocytes were resistant to the anti-lipogenic effects of C13, correlating with a complete loss in AMPK activation, as assessed by the phosphorylation of Thr172 and downstream substrates (Figures 5C–5E). Lipogenesis was, however, still modestly impaired (≈20%) at the highest concentrations of C13 in AMPK-KO hepatocytes. We suspect that this is due to limited, off-target inhibition of acetate-CoA ligase by C13 (Figure S6), as previously reported for nucleotide 5′-alkylphosphates that mimic the transition state (Grayson and Westkaemper, 1988).

**Figure 6:**
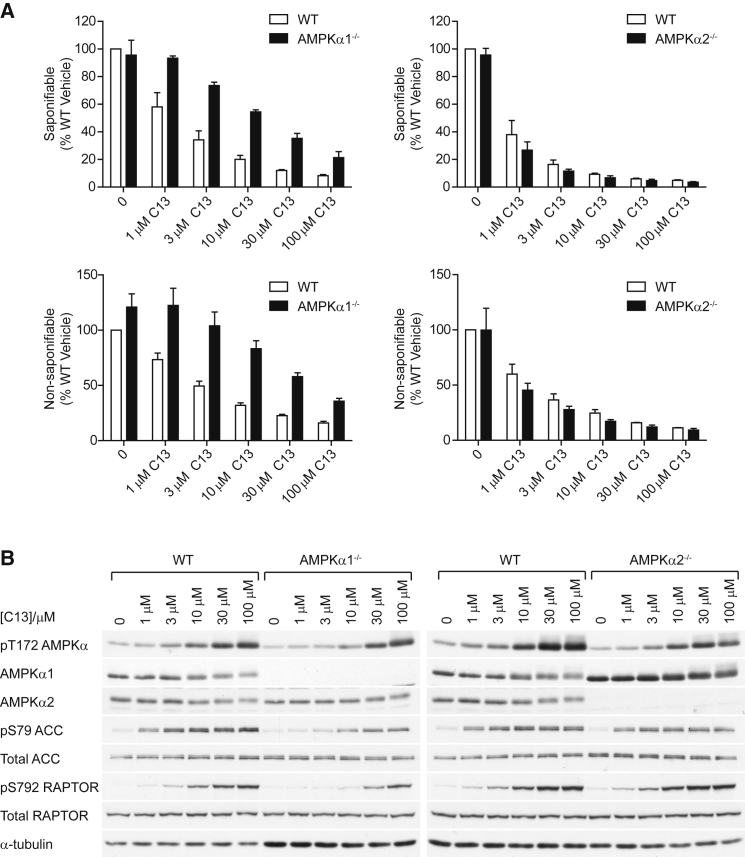
C13 Is Relatively Selective for α1 Complexes in Primary Hepatocytes (A) Hepatocytes from WT, AMPKα1^−/−^, and AMPKα2^−/−^ mice were treated with the indicated concentrations of C13 and labeled with [1-^14^C]acetate for 3 hr. Rates of fatty acid and sterol synthesis were estimated from incorporation into saponifiable (upper graphs) and nonsaponifiable (lower graphs) lipids. Results are expressed as percentage WT vehicle and represent the mean ± SD for three independent experiments. (B) Mouse hepatocytes were incubated with the indicated concentrations of C13 for 3 hr, and lysates were analyzed using western blotting with the indicated antibodies.

Given that α1-containing complexes were more sensitive to C2 in cell-free assays (Figure 1B) and to C13 in intact cells (Figure 4B), we hypothesized that α1-null hepatocytes would be more resistant to C13-induced inhibition of lipid synthesis. Strikingly, there was a shift in concentration dependence for the effect of C13 in α1-KO hepatocytes compared to α2-KO cells; incubation with 1 μM C13 had no effect on the synthesis of saponifiable and nonsaponifiable lipids in α1-KO hepatocytes, whereas the same concentration of C13 reduced lipid synthesis by about 40% in WT cells (Figure 6A). By contrast, α2-KO hepatocytes displayed similar sensitivity to C13 as WT cells (Figure 6A). Thr172 phosphorylation (the antibody detects both α1 and α2) was slightly lower in both α1-KO and α2-KO hepatocytes than in WT cells (Figure 6B). Concentrations of C13 required to promote phosphorylation of Thr172 on AMPKα2, ACC, and Raptor in α1-KO hepatocytes were also higher than those required in WT controls. By contrast, α2-KO hepatocytes displayed similar concentration dependence for C13 on the phosphorylation of AMPK, ACC, and Raptor as WT cells (Figure 6B).

### Effect of C13 on Lipogenesis and AMPK Signaling in AMPKβ1^−/−^ and β2^−/−^ Hepatocytes

**Figure 7:**
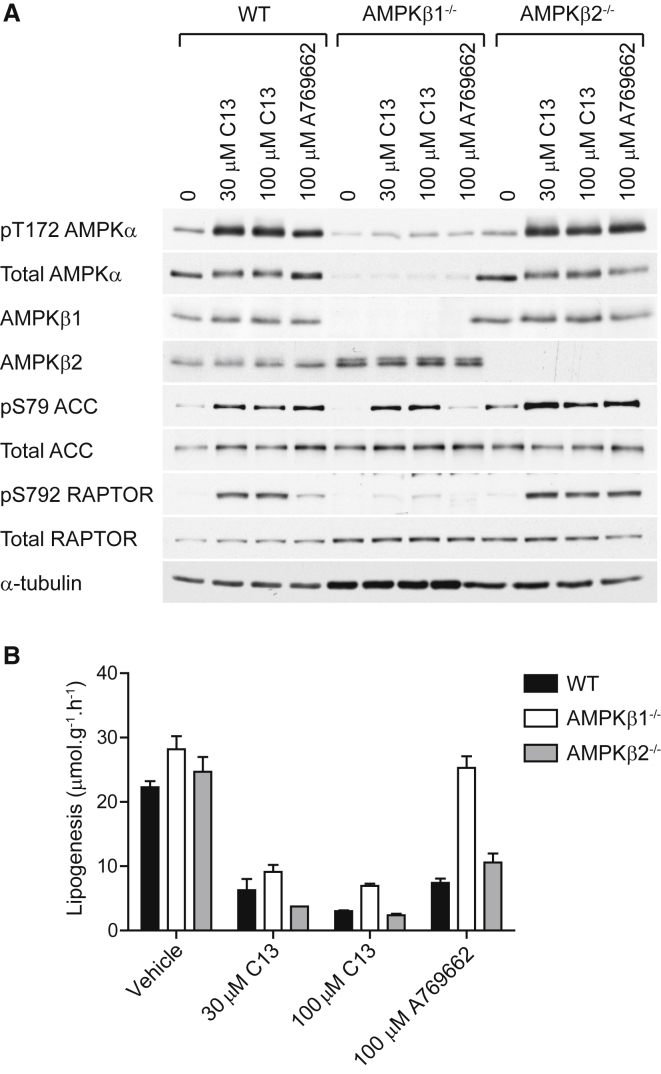
C13 Activates Both β1 and β2 Complexes in Mouse Hepatocytes (A) Hepatocytes from WT, AMPKβ1^−/−^, and AMPKβ2^−/−^ mice were incubated with the indicated concentrations of C13 for 1 hr. A769662 (100 μM) was included as a positive control. Lysates were analyzed using western blotting with the indicated antibodies. (B) Mouse hepatocytes from the indicated genotypes were treated with the indicated concentrations of C13 and labeled with [^3^H]acetate for 4 hr. Lipogenesis was estimated from the incorporation of acetate into total lipids. Results are expressed as the mean acetate incorporated in micromoles per gram-hour ± SD.

Finally, we examined whether C13 requires specific β subunit isoforms to modulate lipid synthesis and AMPK signaling. As previously described (Dzamko et al., 2010), β1 is the predominant isoform in mouse liver, and its deletion resulted in instability and/or degradation of the α subunits, thus reducing Thr172 phosphorylation (although there was some compensatory upregulation of β2) (Figure 7A). By contrast, β2 deletion did not cause a significant reduction in total AMPKα protein or Thr172 phosphorylation (Figure 7A). C13 and A769662 robustly stimulated the phosphorylation of Thr172 and ACC, as well as Raptor, in WT and β2-KO hepatocytes. In β1-KO hepatocytes, C13 modestly stimulated Thr172 phosphorylation, which was sufficient to saturate ACC phosphorylation, although Raptor phosphorylation was only modestly increased. As previously reported (Fullerton et al., 2013; Hawley et al., 2012; Scott et al., 2008), A769662 failed to stimulate AMPK and phosphorylation of ACC and Raptor in β1-KO hepatocytes (Figure 7A). C13 inhibited lipid synthesis to a similar extent in hepatocytes from all genotypes at both at 30 and 100 μM, whereas A769662 failed to suppress lipogenesis in β1-KO hepatocytes, reflecting the known specificity of this compound for β1-containing complexes (Figure 7B).

## Discussion

The original identification of a small-molecule AMPK activator, A769662, provided a key molecular tool to delineate the function of AMPK in intact cells. A unique property of this activator is its selectivity for AMPK complexes containing the β1 subunit, providing researchers an opportunity to study the role of different β subunit isoforms. However, it has limited utility when studying AMPK function in cells or tissues expressing predominantly β2-containing complexes. The publication of a novel activator named C2 (Gómez-Galeno et al., 2010) drew our attention to the strategy of developing cell-permeable AMP mimetics. One concern, as observed with AICAR, was the potential effect of C2 on AMP-regulated enzymes other than AMPK (PFK1, FBP1, and glycogen phosphorylase). However we showed that, unlike ZMP formed from AICAR, C2 does not affect any of these enzymes or several enzymes that use AMP as a substrate. In addition, most kinases (in a panel of 138) were not significantly affected by 10 μM C2, including members of the AMPK-related kinase family or any of the known upstream kinases of AMPK.

Cell-free assays of several AMPK complexes revealed that C2 is a potent allosteric activator of AMPK (EC_50_ of 10–30 nM), which is >20-fold more potent than A769662 (Cool et al., 2006; Göransson et al., 2007) and more than two orders of magnitude more potent than AMP. We also demonstrated an unexpected preference of C2 for α1 complexes. C2 is only a partial agonist for allosteric activation of α2 complexes compared to AMP, and it antagonizes allosteric activation by AMP. By contrast, AMP and C2 are equally effective in the allosteric activation of α1 complexes, and as full agonists, do not exhibit competitive antagonism. Moreover, binding of C2 is much more effective at protecting against the dephosphorylation of Thr172 using α1 rather than α2 complexes, whereas AMP is effective with both isoforms.

C13, a prodrug of C2, activated AMPK in a concentration-dependent manner in isolated mouse hepatocytes (which express both α1 and α2) and inhibited de novo lipid synthesis and fatty acid esterification, effects (at least lipid synthesis) that were abolished in AMPK-KO hepatocytes. C13 was more effective than A769662, which was evident from the inability of A769662 to stimulate robust phosphorylation of Raptor, which in our experience requires a higher threshold of AMPK activity than ACC phosphorylation. We found no change in adenine nucleotide levels during treatment with C13 at concentrations up to 100 μM in primary hepatocytes, showing that the compound does not act indirectly via that mechanism. Furthermore, the preference for α1 was confirmed in intact cells by the poor activation of α2 complexes revealed by isoform-specific immunoprecipitation. This was also demonstrated by the marked increase in IC_50_ for the inhibition of de novo lipogenesis in α1-KO compared to WT hepatocytes, whereas α2 deletion was without significant effect. Similar experiments with β1 or β2 complexes and β1- or β2-KO hepatocytes confirmed that C13 has no preference for β subunit isoforms, either in cell-free assays or in intact cells.

Although we have not identified the precise binding sites used by C2, it is structurally analogous to AMP and is also equally effective with AMP in displacing a GST-γ2 fusion protein from ATP-Sepharose. Moreover, C2 failed to activate AMPK containing a mutant γ2 subunit (R531G), which renders AMPK complexes insensitive to AMP. The side chain of Arg531 interacts with the phosphate group of AMP bound in site 3, so this suggests that C2 binds in that site. These results suggest that C2 uses the same binding sites on the AMPKγ subunit as AMP and ATP, most likely sites 1 and 3 (Xiao et al., 2011). This is also supported by our findings that (1) the effect of C2 on α1 complexes was not additive with that of AMP, that (2) C2 reduced the activation of α2 complexes by AMP, and that (3) the activation of an α1β1γ1 complex by C2 was antagonized by ADP, which also binds to sites 1 and 3. C2 had no effect on the activity of full-length isolated α subunits, unlike nonnucleotide compounds reported to function by disrupting the interaction between the catalytic subunit and the autoinhibitory domain (Li et al., 2013; Pang et al., 2008). In addition, C2 does not produce the biphasic allosteric activation of AMPK by AMP caused by competition of high concentrations of AMP with ATP at the catalytic site (Gowans et al., 2013).

Assuming that C2 binds exclusively to the nucleotide sites on the γ subunit, its ability to discriminate between α1 and α2 complexes was unexpected, so we sought to identify the mechanism underlying this. The γ subunit has four potential nucleotide-binding sites, but one (site 2) is unused, leaving three (sites 1, 3, and 4) where adenine nucleotides can bind. In the structure for an active α1β2γ1 complex (Xiao et al., 2011), the α linker that connects the AID and CTD wraps around one face of the γ subunit, contacting AMP bound in site 3 (Xiao et al., 2011). Interestingly, there is relatively low conservation of sequence between α1 and α2 within the α linker, and this has been exploited to generate isoform-specific antibodies. Based on the original model (Xiao et al., 2011), it was proposed that a region in the α linker termed the α hook (R384-N393 in rat α1, P54645) interacted with AMP in site 3. We therefore hypothesized that differences in the interaction between the α1 and α2 hook and C2 bound in site 3 might be responsible for the ability of C2 to discriminate between α subunit isoforms. However, substitution of the α hook region in a complex between an α2/α1 chimera and β1 and γ1 had no impact on the sensitivity to C2, although replacement of a more extended region of the α linker fully restored sensitivity to the allosteric activation and protection of Thr172 phosphorylation by C2. The likely explanation for this anomaly came when it was suggested (Chen et al., 2013; Xin et al., 2013) that the original assignment of amino acid sequence to electron density in the α hook region (Xiao et al., 2011) may have been incorrect. Very recently this change has been accepted by the original authors (Xiao et al., 2013), resulting in the replacement of the original atomic coordinates in the Protein Data Bank (PDB ID 2Y94) with a revised version (4CFH). This model suggests that the region from 343 to 353 in human α1 (Q13131), termed α-regulatory subunit-interacting motif-1 (α-RIM1), associates with the unoccupied site 2, whereas the region from 369 to 379 (α-RIM2) associates with AMP bound in site 3 (Xin et al., 2013). Using our chimeric α2/α1 loop complex, in which a more extended region of the α1 linker (including α-RIM2 and the former α hook sequence, but excluding α-RIM1) was used to replace the equivalent region in an α2β1γ1 complex, we observed full allosteric activation and protection against Thr172 dephosphorylation. These results support the revised model and also strongly suggest that the different sequences of α1 and α2 in the α-RIM2 region cause differing interactions with C2 bound in site 3, leading to the selectivity of C2/C13 for α1 versus α2 complexes. Our results also support the view that C2 binds at site 3 and that this affects both allosteric activation and protection against the dephosphorylation of Thr172. Xiao et al. (2011) previously argued that site 1 was the important binding site for allosteric activation by AMP, whereas Chen et al. (2012) provided evidence in favor of the importance of sites 3 and 4. Our results support an important role for site 3 while not excluding additional roles of sites 1 and 4.

In summary, we report the detailed characterization and potential mechanism of action of an AMP-mimetic but α1-selective AMPK activator that, unlike AICAR or ZMP, is completely selective for AMPK compared to other AMP-regulated or -metabolizing enzymes. Although the preference for α1-containing complexes may limit its use for some indications, this shows that it is possible to develop α isoform-specific activators, along with the β isoform-selective activators typified by A769662. Recently, Scott et al. (2014b) reported a small-molecule activator of AMPK (JJO1) that activated AMPKα1- and AMPKα2-containing complexes independently of the β subunit CBD but was inactive with γ3. A more complete understanding of the mechanism by which small molecules activate AMPK may facilitate the design of additional AMPK activators that could be used to treat patients with metabolic disorders.

## Significance

**AMP-activated protein kinase (AMPK) is a central energy sensor and regulator of metabolic homeostasis. The activation of AMPK provides desirable therapeutic effects in metabolic disorders such as type 2 diabetes. However, there is currently no direct AMPK activator available for the treatment of metabolic disorders. Only a handful of small molecules have been reported to directly stimulate AMPK with no defined mechanism of action mode elaborated except for A769662, which stimulates β1-containing complexes. We performed an extensive characterization of a recently identified AMPK activator, a nucleotide mimetic, termed Compound 2 (C2) and its prodrug C13. We observed that C2 stimulates AMPK at least 20-fold more potently than A769662 in cell-free assays, with absolute specificity over other AMP-regulated or -metabolizing enzymes. We also found that C2 stimulates AMPK by mimicking both effects of AMP, allosteric activation and inhibition of dephosphorylation via protein phosphatase (PP2C). Strikingly, we identified that C2 preferentially stimulates α1-containing complexes and identified a sequence located in the C-terminal region of α1, outside the catalytic domain, which confers this specificity. The selectivity of the compound for α1 complexes in cell-free assays was consistent with the ability of the cell-permeable prodrug C13 to potently inhibit hepatic lipogenesis in primary mouse hepatocytes, which was reversed in AMPKα1-deficient hepatocytes. This demonstrates that it is possible to develop isoform-selective compounds outside the β subunit carbohydrate-binding-module-dependent compounds, typified by A769662. A more complete understanding of the mechanism by which small molecules activate AMPK may also facilitate the design of novel AMPK activators that could be used to treat patients with metabolic disorders.**

## Experimental Procedures

### Animals

Animal studies were approved by the University of Dundee ethics committee and performed under a UK Home Office project license. All animals were maintained on a 12/12 hr light/dark cycle and had free access to standard chow and water. AMPKα1^−/−^, AMPKα2^−/−^ and liver AMPK-null (AMPKα1^−/−^ and liver-specific AMPKα2^−/−^) mice were generated and bred, as previously described (Foretz et al., 2010; Jørgensen et al., 2004; Viollet et al., 2003). Experiments using AMPKα-null models were performed under the approval of the ethics committee from University Paris Descartes (no. CEEA34.BV.157.12) and a French authorization to experiment on vertebrates (no.75-886) in accordance with the European guidelines. AMPKβ1^−/−^ and AMPKβ2^−/−^ mice were generated as previously described (Dzamko et al., 2010; Steinberg et al., 2010), and experiments were conducted under the approval of the McMaster University animal ethics research board.

### AMPK Assay

AMPK phosphotransferase activity was assayed in reactions (50 μl) containing 50 mM HEPES, pH 7.4, 10 mM MgCl_2_, 0.1 mM EGTA, 1 mM DTT, 0.01% BRIJ-35, 100 μM [γ-^32^P]ATP (∼250 CPM⋅pmol^−1^) and 200 μM AMARA (AMARAASAAALARRR). Reactions were started by the addition of AMPK (5 mU), incubated for 20 min at 30°C and quenched by spotting onto P81 and immersion in 75 mM H_3_PO_4_. Washed filters were dried and [^32^P] incorporation determined by Cherenkov counting. AMPK activity in cell extracts was determined by immunoprecipitation with AMPKα1 or AMPKα2 antibodies from 50 μg material, as previously described (Hunter et al., 2011). Results are expressed as picomoles P_*i*_ incorporated per minute-milligram or the fold increase in activity compared to controls in the absence of the compound and were fitted to the following equation:

$$v=v_{o}+\left\{\frac{\left(v_{\max}-v_{o}\right){\left[A\right]}^{h}}{K_{a}^{h}+{\left[A\right]}^{h}}\right\},$$

where *v* is the velocity, *v*_*o*_ is the velocity in the absence of compound, [*A*] is the concentration of activator, *K*_*a*_ is the concentration of activator that increases velocity to 50% maximal stimulated activity, and *h* is the Hill coefficient.

### Phosphatase Protection Assay

AMPKα1β1γ1 or α2β1γ1 was dephosphorylated in vitro with PP2Cα in 50 mM HEPES pH 7.4, 10 mM MgCl_2_, 0.1 mM EGTA, 0.03% BRIJ-35, 1 mM DTT, and the indicated compounds for 15 min at 30°C. Mg^2+^ was omitted from the negative control. Reactions were terminated by 20-fold dilution and storage on ice. AMPK activity was assayed under standard conditions in the presence of saturating AMP (200 μM). The results are expressed as percentage activity of the negative control. In control experiments, PP2Cα activity was determined in reactions containing 50 mM TES pH 7.4, 0.1 mM EGTA, 25 mM MgCl_2_, 0.01% BRIJ-35, 0.02% (v/v) 2-mercaptoethanol, and 100 μM EFLR(pT)SCGS (168–176 AMPKα2). Liberated phosphate was determined using malachite green (Baykov et al., 1988).

### Lipid Synthesis

Lipid synthesis was determined by labeling adherent cultures of primary hepatocytes with [1-^14^C]acetate. After overnight culture, hepatocytes (5 × 10^5^) were washed with warm PBS and allowed to rest for 3 hr in fresh M199. Cells were treated with vehicle (0.1% DMSO) or the indicated compounds and labeled with 1 mCi⋅mmol^−1^ [1-^14^C]acetate for 3 hr. Cells were washed with ice-cold PBS, gently scraped into 0.5 ml PBS and saponified in methanolic KOH at 80°C for 1 hr. Nonsaponifiable and saponifiable lipids were extracted with petroleum ether before and after acidification with HCl. Lipid fractions were washed with 5% HAc, dried using N_2_, and dissolved in scintillant for the determination of [^14^C] incorporation. Lipogenesis in hepatocytes from AMPKβ1/β2-KO mice was determined in media containing [^3^H]acetate (0.2 mCi⋅mmol^−1^). After 4 hr labeling, total lipids were extracted using the method of Bligh and Dyer (1959), and incorporated [^3^H] was determined by scintillation counting. The results are expressed as micromoles acetate incorporated per gram-hour.

### Fatty Acid Esterification

Fatty acid esterification was determined by labeling primary hepatocytes with [9,10-^3^H(N)]palmitate. After overnight culture, hepatocytes (5 × 10^5^) were washed with warm PBS and allowed to rest for 3 hr in fresh M199 containing 0.5 mM carnitine. Cells were treated with vehicle (0.1% DMSO) or the indicated compounds for 30 min and labeled with 1 mCi⋅mmol^−1^ [9,10-^3^H(N)]palmitic acid (0.5 mM palmitic acid, 1.34% BSA = 2.5:1 C16:0/BSA) for an additional 1 hr. Cells were washed with ice-cold PBS, lipids extracted using the method of Bligh and Dyer, and neutral lipids were resolved on TLC plates (Partisil K6) in 70:30:1 petroleum ether:diethyl ether:acetic acid. Lipids were stained with iodine vapor and triglyceride eluted from TLC scrapings with 1:1 ethanol:Triton X-100, and the incorporation of [^3^H] was determined by scintillation counting.

## Supplemental Information

Document S1. Supplemental Experimental Procedures and Figures S1–S6

Document S2. Article plus Supplemental Information
