# Supplementary material for: Mechanism of Action of Compound-13: An α1-Selective Small Molecule Activator of AMPK
Source: Chem Biol. 2014 Jul 17;21(7):866–79. doi: 10.1016/j.chembiol.2014.05.014 (PMC4104029; doi:10.1016/j.chembiol.2014.05.014)
Supplement: Document S1. Supplemental Experimental Procedures and Figures S1–S6 [file mmc1.pdf]

Chemistry & Biology, Volume *21*

Supplemental Information

Mechanism of Action of Compound-13:

An α1-Selective Small Molecule Activator of AMPK

Roger W. Hunter, Marc Foretz, Laurent Bultot, Morgan D. Fullerton, Maria Deak, Fiona A. Ross, Simon A. Hawley, Natalia Shpiro, Benoit Viollet, Denis Barron, Bruce E. Kemp, Gregory R. Steinberg, D. Grahame Hardie, and Kei Sakamoto

Figure S1

A - AMPK complex preparations

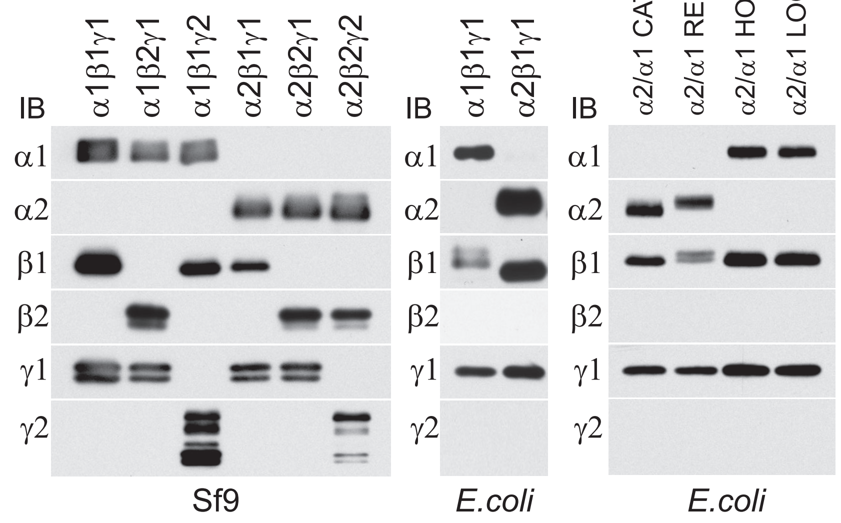

B

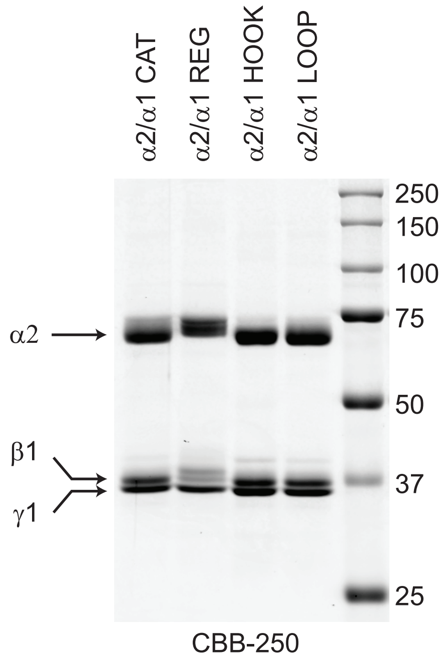

Figure S2

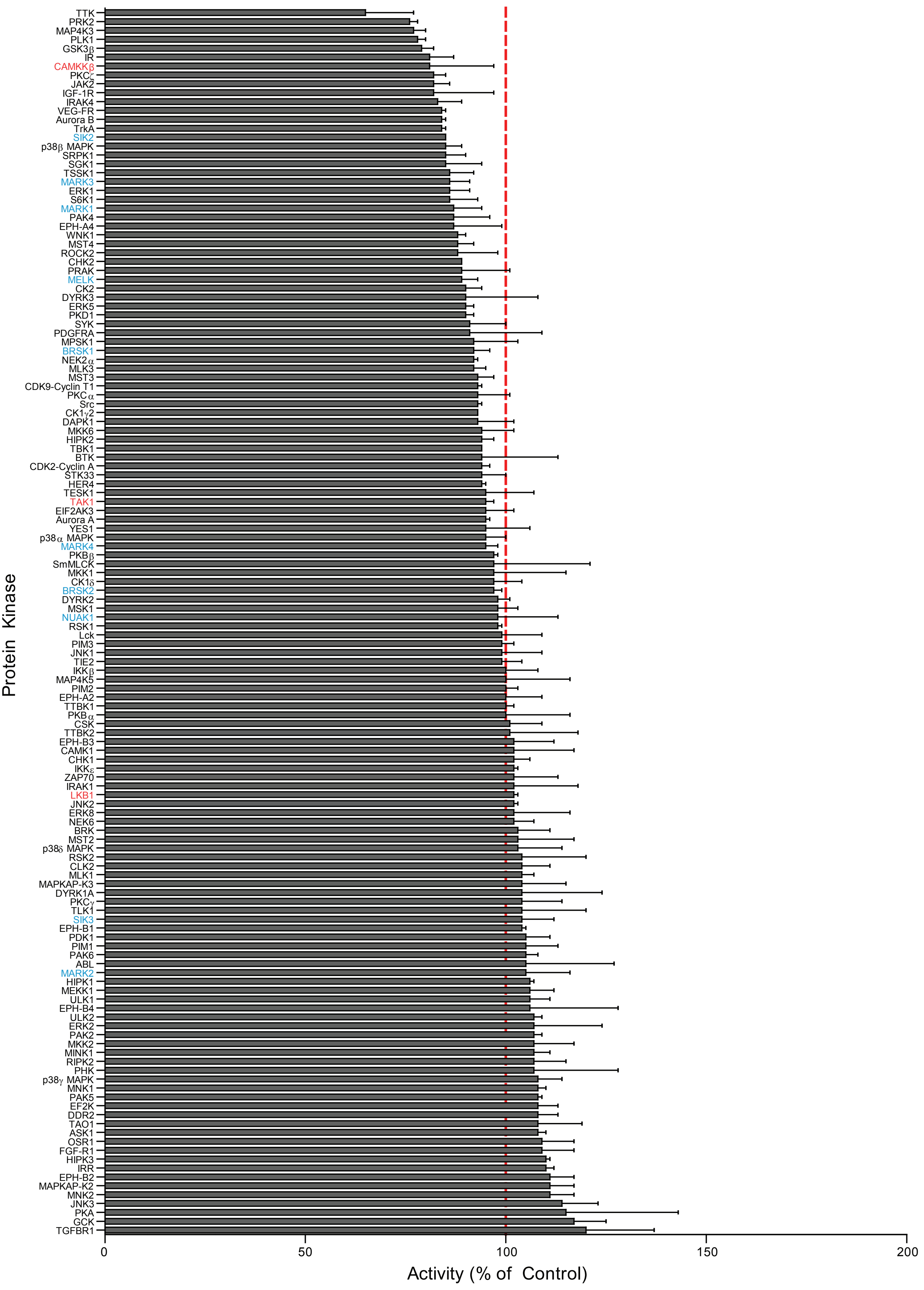

Protein Kinase
TTK
PRK2
MAP4K3
PLK1
GSK3β
IR
CAMKKβ
PKCζ
JAK2
IGF-1R
IRAK4
VEG-FR
Aurora B
TrkA
SIK2
p38β MAPK
SRPK1
SGK1
TSSK1
MARK3
ERK1
S6K1
MARK1
PAK4
EPH-A4
WNK1
MST4
ROCK2
CHK2
PRAK
MELK
CK2
DYRK3
ERK5
PKD1
SYK
PDGFRA
MPSK1
BRSK1
NEK2α
MLK3
MST3
CDK9-Cyclin T1
PKCα
Src
CK1γ2
DAPK1
MKK6
HIPK2
TBK1
BTK
CDK2-Cyclin A
STK33
HER4
TESK1
TAK1
EIF2AK3
Aurora A
YES1
p38α MAPK
MARK4
PKBβ
SmMLCK
MKK1
CK1δ
BRSK2
DYRK2
MSK1
NUAK1
RSK1
Lck
PIM3
JNK1
TIE2
IKKβ
MAP4K5
PIM2
EPH-A2
TTBK1
PKBα
CSK
TTBK2
EPH-B3
CAMK1
CHK1
IKKε
ZAP70
IRAK1
LKB1
JNK2
ERK8
NEK6
BRK
MST2
p38δ MAPK
RSK2
CLK2
MLK1
MAPKAP-K3
DYRK1A
PKCγ
TLK1
SIK3
EPH-B1
PDK1
PIM1
PAK6
ABL
MARK2
HIPK1
MEKK1
ULK1
EPH-B4
ULK2
ERK2
PAK2
MKK2
MINK1
RIPK2
PHK
p38γ MAPK
MNK1
PAK5
EF2K
DDR2
TAO1
ASK1
OSR1
FGF-R1
HIPK3
IRR
EPH-B2
MAPKAP-K2
MNK2
JNK3
PKA
GCK
TGFBR1
0
50
100
150
200
Activity (% of Control)

Figure S3

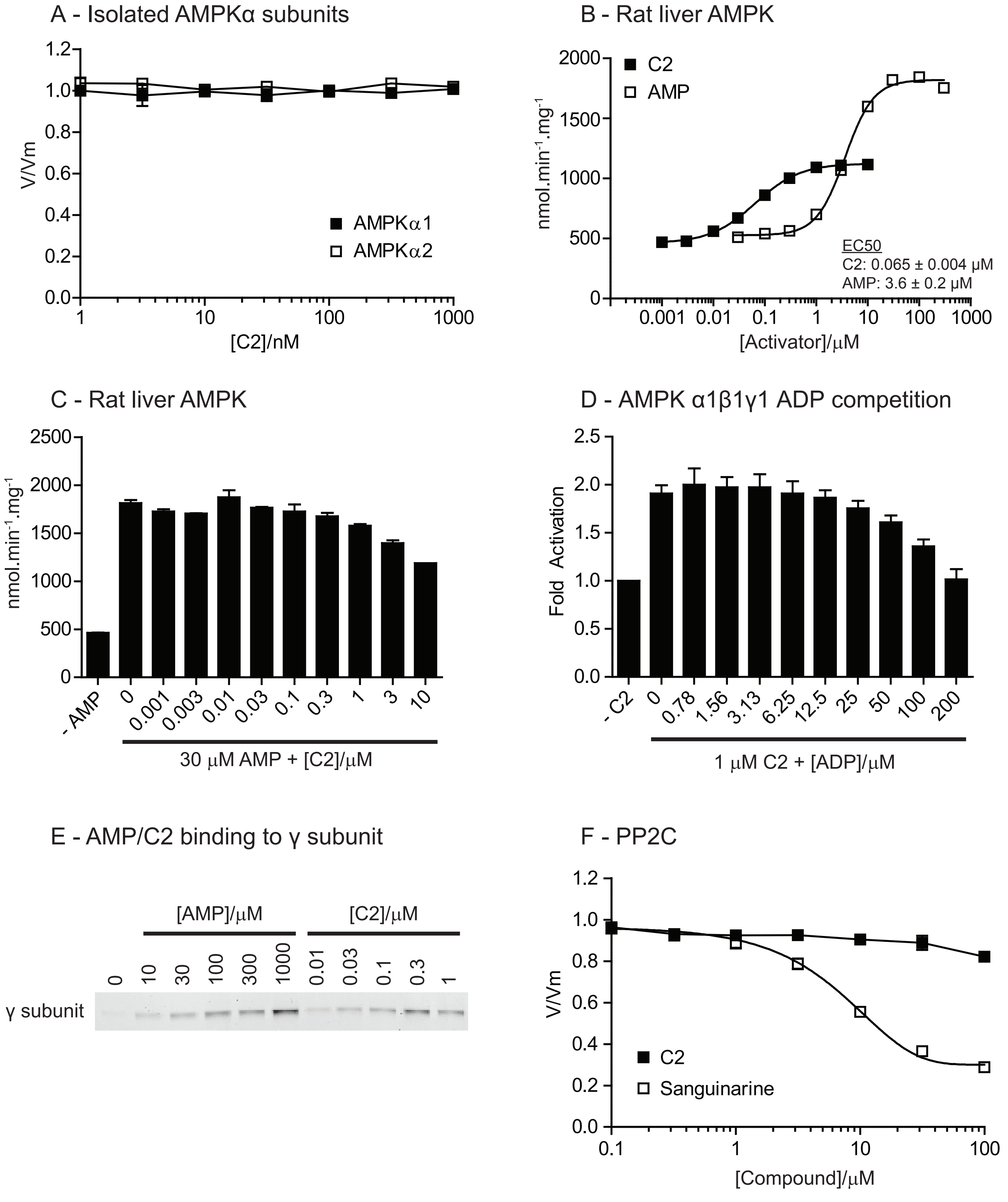
A - Isolated AMPKα subunits
V/Vm
[C2]/nM
AMPKα1
AMPKα2
B - Rat liver AMPK
nmol.min-1.mg-1
[Activator]/μM
C2
AMP
EC50
C2: 0.065 ± 0.004 μM
AMP: 3.6 ± 0.2 μM
C - Rat liver AMPK
nmol.min-1.mg-1
- AMP
30 μM AMP + [C2]/μM
D - AMPK α1β1γ1 ADP competition
Fold Activation
- C2
1 μM C2 + [ADP]/μM
E - AMP/C2 binding to γ subunit
[AMP]/μM
[C2]/μM
γ subunit
F - PP2C
V/Vm
[Compound]/μM
C2
Sanguinarine

Figure S4

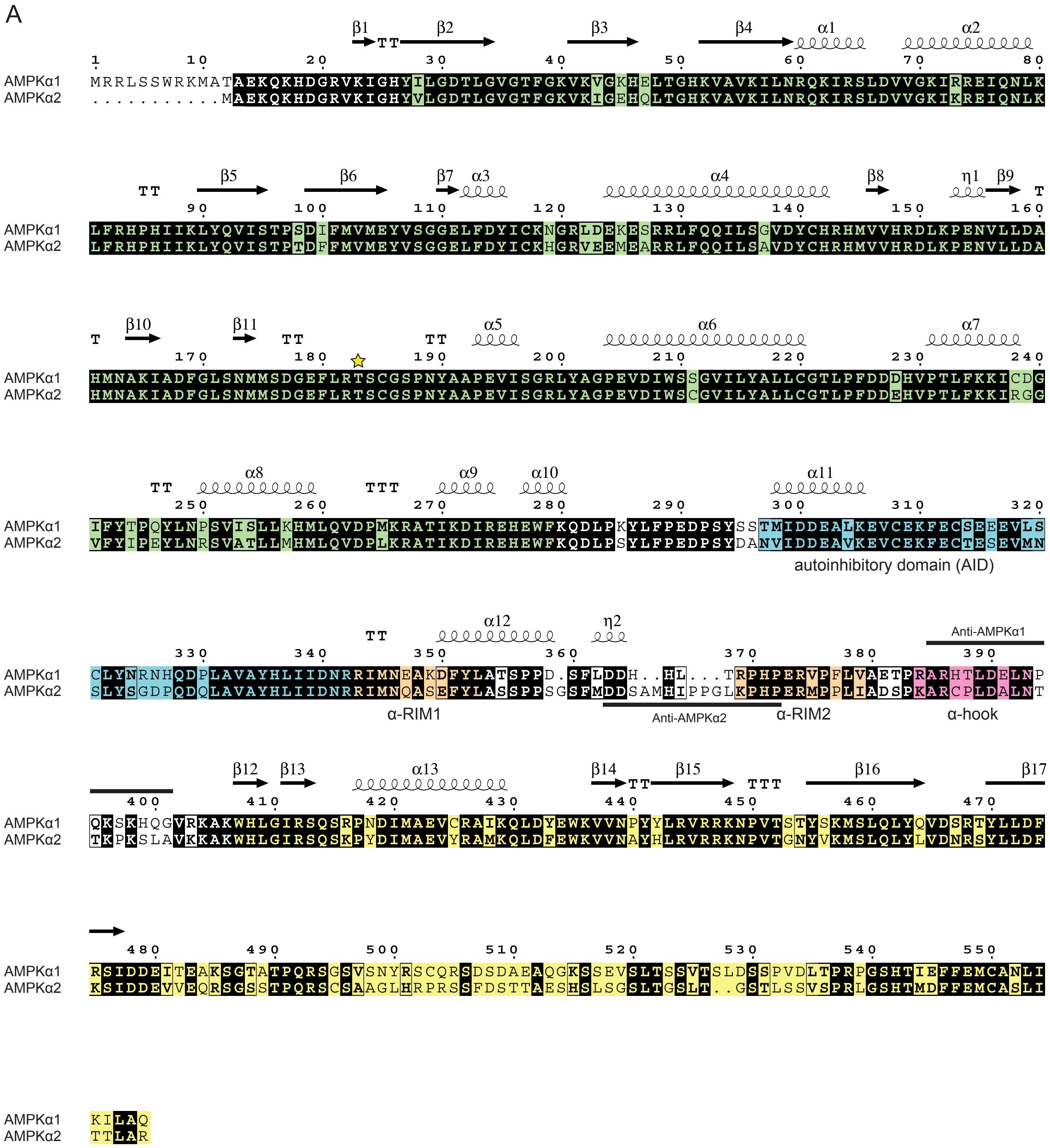

A
β1 TT β2 β3 β4 α1 α2
AMPKα1 MRRLSSWRKMATAEKQKHDGRVKIGHYILGDTLGVGTFGKVKVGKHELTGHKVAVKILNRQKIRSLDVVGKIRREIQNLK
AMPKα2 ...........MAEKQKHDGRVKIGHYVLGDTLGVGTFGKVKIGEHQLTGHKVAVKILNRQKIRSLDVVGKIKREIQNLK
TT β5 β6 β7 α3 α4 β8 η1 β9 T
AMPKα1 LFRHPHIIKLYQVISTPSDIFMVMEYVSGGELFDYICKNGRLDEKESRRLFQQILSGVDYCHRHMVVHRDLKPENVLLDA
AMPKα2 LFRHPHIIKLYQVISTPTDFFMVMEYVSGGELFDYICKHGRVEEMEARRLFQQILSAVDYCHRHMVVHRDLKPENVLLDA
T β10 β11 TT TT α5 α6 α7
AMPKα1 HMNAKIADFGLSNMMSDGEFLRTSCGSPNYAAPEVISGRLYAGPEVDIWSSGVILYALLCGTLPFDDDHVPTLFKKICDG
AMPKα2 HMNAKIADFGLSNMMSDGEFLRTSCGSPNYAAPEVISGRLYAGPEVDIWSCGVILYALLCGTLPFDDEHVPTLFKKIRGG
TT α8 TTT α9 α10 α11
AMPKα1 IFYTPQYLNPSVISLLKHMLQVDPMKRATIKDIREHEWFKQDLPKYLFPEDPSYSSTMIDDEALKEVCEKFECSEEEVLS
AMPKα2 VFYIPEYLNRSVATLLMHMLQVDPLKRATIKDIREHEWFKQDLPSYLFPEDPSYDANVIDDEAVKEVCEKFECTESEVMN
autoinhibitory domain (AID)
TT α12 η2 Anti-AMPKα1
AMPKα1 CLYNRNHQDPLAVAYHLIIDNRRIMNEAKDFYLATSPPD.SFLDDH..HL...TRPHPERVPFLVAETPRARHTLDELNP
AMPKα2 SLYSGDPQDQLAVAYHLIIDNRRIMNQASEFYLASSPPSGSFMDDSAMHIPPGLKPHPERMPPLIADSPKARCPLDALNT
α-RIM1
Anti-AMPKα2
α-RIM2
α-hook
β12 β13 α13 β14 TT β15 TTT β16 β17
AMPKα1 QKSKHQGVRKAKWHLGIRSQSRPNDIMAEVCRAIKQLDYEWKVVNPYYLRVRRKNPVTSTYSKMSLQLYQVDSRTYLLDF
AMPKα2 TKPKSLAVKKAKWHLGIRSQSKPYDIMAEVYRAMKQLDFEWKVVNAYHLRVRRKNPVTGNYVKMSLQLYLVDNRSYLLDF
AMPKα1 RSIDDEITEAKSGTATPQRSGSVSNYRSCQRSDSDAEAQGKSSEVSLTSSVTSLDSSPVDLTPRPGSHTIEFFEMCANLI
AMPKα2 KSIDDEVVEQRSGSSTPQRSCSAAGLHRPRSSFDSTTAESHSLSGSLTGSLT..GSTLSSVSPRLGSHTMDFFEMCASLI
AMPKα1 KILAQ
AMPKα2 TTLAR

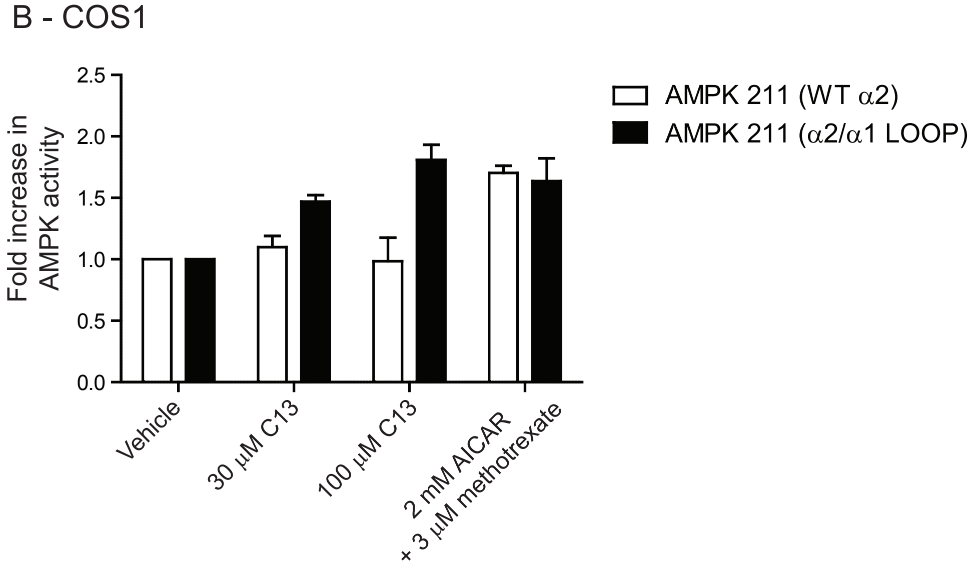

B - COS1
Fold increase in AMPK activity
2.5
2.0
1.5
1.0
0.5
0.0
Vehicle
30 μM C13
100 μM C13
2 mM AICAR + 3 μM methotrexate
AMPK 211 (WT α2)
AMPK 211 (α2/α1 LOOP)

Figure S5

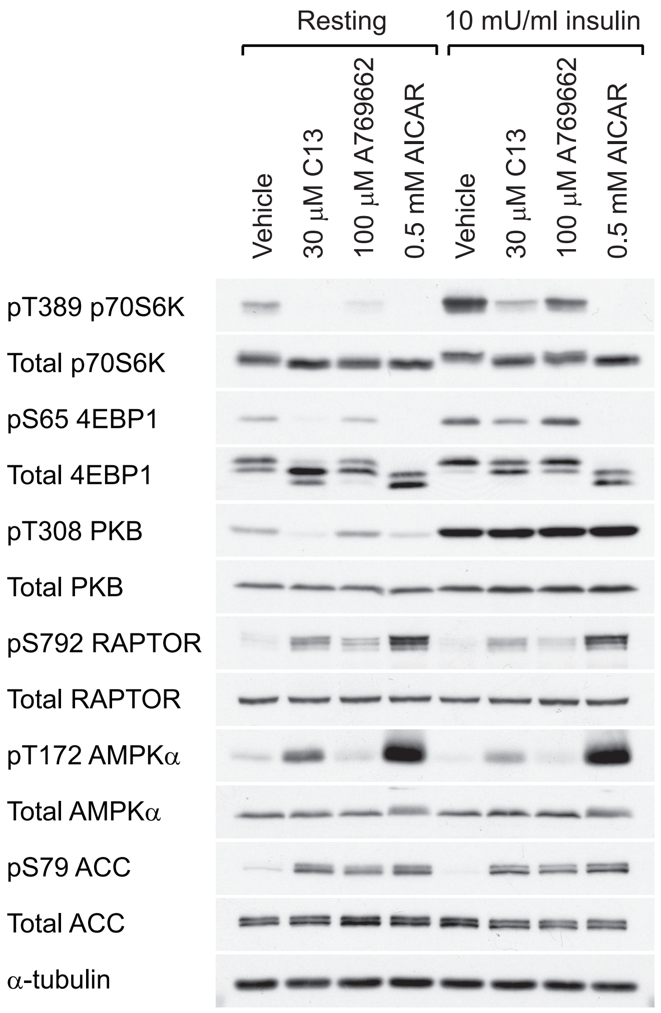
Resting
10 mU/ml insulin
Vehicle
30 μM C13
100 μM A769662
0.5 mM AICAR
Vehicle
30 μM C13
100 μM A769662
0.5 mM AICAR
pT389 p70S6K
Total p70S6K
pS65 4EBP1
Total 4EBP1
pT308 PKB
Total PKB
pS792 RAPTOR
Total RAPTOR
pT172 AMPKα
Total AMPKα
pS79 ACC
Total ACC
α-tubulin

Figure S6

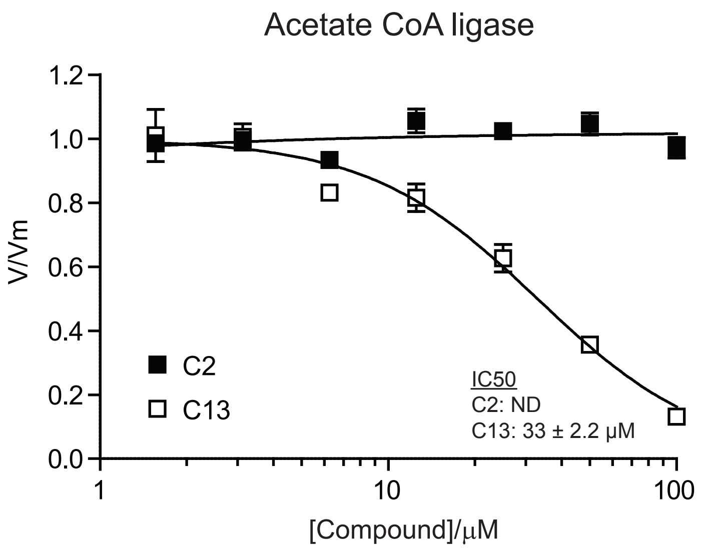

Acetate CoA ligase
1.2
1.0
0.8
0.6
0.4
0.2
0.0
V/Vm
C2
C13
IC50
C2: ND
C13: 33 ± 2.2 μM
1
10
100
[Compound]/μM

Supplemental Figure Legends:

Figure S1, related to Figures 1-3. Subunit composition of AMPK preparations used in this study. (A) Heterotrimeric AMPK complexes (10 ng) produced in both Sf9 (Sigma and Signalchem) and *E.coli* (in our laboratory) were analysed by Western blotting using isoform specific antibodies. Note that both the α2/α1-HOOK and α2/α1-LOOP chimeras are no longer recognised by the anti-α2 antibody as the epitope has been replaced (see Fig S4). (B) Heterotrimeric AMPK α2/α1 chimeric complexes expressed in *E.coli* (2 μg) were separated by SDS-PAGE and stained with Coomassie G-250. Arrows indicate the position of the AMPKα, β and γ subunits.

Figure S2, related to Figure 1. C2 has negligible effects on a panel of 138 protein kinases. Kinase inhibition screen was performed by the International Centre for Kinase profiling (Dundee). Assays were performed in duplicate using 10 µM C2 and are representative of two independent screens. Results are shown in rank order as mean % activity compared with controls in the absence of compound ± SD. Members of the AMPK-related kinase family are highlighted in blue whereas known upstream kinases of AMPK are shown in red.

Figure S3, related to Figure 2. C2 is a partial agonist of rat liver AMPK and is antagonised by ADP but has no effect on isolated AMPKα subunits. (A) Full-length human AMPKα1 and α2 subunits were prepared in *E.coli* and activated with CAMKK as described in Methods. Isolated AMPKα subunits were assayed under standard conditions in the presence of C2 (0 - 1000 nM). Results are expressed normalised to maximal activity in the absence of ligand and represent the average ± SD of three independent experiments on a single preparation of kinase. (B) Rat liver purified AMPK (containing α1 and α2 subunits in complex with β1γ1) was assayed for activation by AMP and C2 in the presence of 0.2 mM ATP and 0.2 mM AMARA as described in Methods. Results are expressed as mean P_i incorporated in $nmol.min^{-1}.mg^{-1}$ ± SD. (C) Rat liver AMPK was assayed under standard conditions in the presence of saturating AMP (30 µM) and increasing concentrations of C2 (0 - 10 µM). (D) Recombinant AMPK α1β1γ1 was assayed under standard conditions in the presence of 1 µM C2 and increasing concentrations of ADP (0 - 200 µM). Results are expressed as fold increase in activity relative to controls in the absence of compound ± SD. (E) C2 and AMP displace a GST-AMPKγ2 subunit fusion from ATP-γ-Sepharose. A purified C-terminal fragment of AMPKγ2 bearing CBS domains 1-4 was absorbed onto ATP-Sepharose. Immobilised GST-AMPKγ2 was dispensed into tubes and eluted with the indicated ligands (C2 or AMP). The presence of eluted AMPKγ2 was analysed by SDS-PAGE and staining with colloidal Coomassie. (F) Recombinant PP2Cα was assayed by monitoring the release of phosphate from 100 µM EFLR(pT)SCGS in the presence of increasing concentrations of C2 (0 – 100 µM). The plant alkaloid, sanguinarine was included as a positive control for inhibition of phosphatase activity.

Figure S4, related to Figure 3. Sequence alignment of AMPKα1 and α2 subunits highlighting key regulatory elements. Global pairwise alignment of human AMPKα1 (Q13131) and AMPKα2 (P54646) performed using the Needleman-Wunsch algorithm

(Needleman and Wunsch, 1970) and edited using ESPript (Gouet, et al., 2003). Secondary structure was derived from PDBID 2Y94. The phosphotransferase domain is shaded green (T-loop phosphorylation site is indicated with a star), the autoinhibitory domain in blue and the C-terminal domain in yellow. Regulatory elements in the α-linker are also indicated: α-hook in pink and α-RIMs in orange. Black lines indicate the position of the epitopes recognised by α-subunit specific antibodies used in this study. (B) COS-1 cells were seeded into 6-well plates and transiently transfected with pCMV-AMPKβ1, pCMV-AMPKγ1 and either pCMV-FLAG-AMPKα2 WT or pCMV-FLAG-AMPKα2/α1 LOOP chimera using FuGENE HD. After 24 h cells were treated with the indicated compounds for 1 h and kinase activity determined in anti-FLAG immunoprecipitates under standard conditions. Results are normalised to activity in cells treated with vehicle and represent mean ± SD of three independent experiments.

Figure S5, related to Figure 4. C13 inhibits mTOR signalling in mouse hepatocytes. Mouse hepatocytes were incubated with the indicated compounds in the absence (Resting) or presence of 10 mU/ml insulin for 60 min. Lysates were analysed by Western blotting using the indicated antibodies.

Figure S6, related to Figure 5. C13 but not C2 inhibits Acetate CoA ligase (EC 6.2.1.1). Yeast acetate CoA ligase (Sigma) was assayed in reactions containing 50 mM TES pH 7.4, 0.1 mM EDTA, 5 mM $MgCl_2$, 4 mM ATP, 0.2 mM coenzyme A, 5 mM DTT and increasing concentrations of C2 and C13 (0 – 100 μM). The formation of pyrophosphate (PP_i) was monitored by reduction of PP_i-molybdate complexes as described in Methods. Results are expressed relative to control reactions in the absence of compound and represent the mean ± SD of three independent experiments on one preparation of enzyme.

Supplemental Experimental Procedures:

Materials. Compounds **2** and **13** were synthesised by PeakDale Molecular and Natalia Shpiro (MRC-PPU, Dundee), respectively, as described previously (Gomez-Galeno, et al., 2010). A769662 was from Selleck Chemicals and AICAR from Toronto Research Chemicals. Mouse FBP1, PYGM and PFK1 (muscle isoform) were cloned, expressed and purified from *E.coli* using standard techniques. AMPD was purified from chicken muscle (Smiley, et al., 1967). PP2Cα expressed in *E.coli* was from the Division of Signal Transduction Therapy (DSTT, Dundee). Peptides were synthesised by GL Biochem (Shanghai). [γ-^{32}P]-ATP, [1-^{14}C]-acetic acid and [9,10-^{3}H(N)]-palmitic acid were from PerkinElmer. pS79 ACC1 (#3661), ACC1 (#3676), pS792 RAPTOR (#2083), RAPTOR (#2280), pS555 ULK1 (#5869), ULK1 (#8054), pT172 AMPKα (#2535), AMPKα (#2532), AMPKβ1 (#4182), AMPKβ2 (#4148), pT389 p70S6K (#9234), p70S6K (#2708), pS65 4EBP1 (#9456), 4EBP1 (#9452) and LKB1 (#3047) antibodies were purchased from Cell Signaling Technology. Anti-AMPKα1 (07-350) and AMPKα2 (07-363) used for immunoblotting were from Merck-Millipore. Anti-AMPKγ1 was from Origene (clone Y308). Anti-AMPKγ2 was from Epitomics (S1562). Anti-tubulin (B-5-1-2), anti-FLAG (F7425) and anti-FLAG M2 agarose were from Sigma. Anti-HA (HA.11) was from Covance Research Products. AMPKα1/2 antibodies used for immunoprecipitation kinase assays were described previously (Sakamoto, et al., 2006). Phosphocellulose P81 and ECL reagent were from GE Healthcare. HRP-conjugated secondary antibodies were from Jackson Immunoresearch. Human insulin (Actrapid) was from Novo Nordisk. Mouse embryonic fibroblasts (MEF) from LKB1$^{+/+}$ and LKB1$^{-/-}$ mice were generated as described (Hawley, et al., 2003) and were kindly donated by Tomi Makela (University of Helsinki). MEFs were maintained in DMEM containing 10 % (v/v) foetal calf serum (FCS), 100 U/ml penicillin G and 100 µg/ml streptomycin (Hawley, et al., 2003). COS-1 cells were obtained from the ATCC and maintained in DMEM containing 10 % (v/v) FCS. Cells were transfected at 70 % confluency using FuGENE HD (Promega) according to the manufacturer's recommendations. All other materials unless otherwise indicated were from Sigma.

AMPK preparations. Human AMPK complexes (α1β1γ1, α1β2γ1, α1β1γ2, α2β1γ1, α2β2γ1 and α2β2γ2) expressed in *Spodoptera frugiperda* were purchased from Sigma and Signalchem. AMPK from Signalchem was activated by a proprietary cell treatment prior to purification, whereas the material from Sigma was activated by conventional *in vitro* phosphorylation (identity of the kinase is proprietary). Human AMPK α1β1γ1, α2β1γ1 WT and α1/α2 chimeras were expressed in *E. coli* using tricistronic constructs and activated with CAMKKβ as previously described (Neumann, et al., 2003). Isolated human AMPKα subunits were expressed as N-terminal GST-fusions in *E.coli* and activated as described for heterotrimeric complexes. The GST tag was removed using HRV-3C protease prior to assay. A C-terminal fragment of AMPKγ2 bearing CBS domains 1-4 (247-569) was expressed in *E.coli* as an N-terminal GST fusion in LB media supplemented with 1 M sorbitol and 2.5 mM betaine and induced with 1 mM IPTG for 16 h at 20°C. Cells were disrupted by grinding using a mortar and pestle and lysed in 50 mM tris pH 8.5, 0.5 M NaCl, 0.5 M sucrose, 1 mM EGTA, 1 mM EDTA and 1 mM DTT. GST-AMPKγ2 was isolated from clarified lysate using a 5 ml glutathione Sepharose fast flow column (GE Healthcare) and eluted in 50 mM tris pH

8, 0.2 M NaCl and 20 mM glutathione. Rat liver AMPK (a mixture of α1 and α2 complexes with β1γ1) was purified to the gel filtration step (Hawley, et al., 1996). Human AMPK α1β1γ2 WT and associated mutants were prepared in COS1 cells by transient transfection with AMPKα1, HA-AMPKβ1 and FLAG-AMPKγ2 constructs in pCMV using FuGENE HD. After 36 h, cells were harvested as described for Western blotting (see below) and heterotrimeric complexes captured on FLAG-M2 agarose for 2 h at 4°C. Resin was washed extensively with lysis buffer and equilibrated with HBS (50 mM HEPES pH 7.4, 150 mM NaCl). Preparations were activated with CAMKKβ for 30 min at 30°C in the presence of 10 mM $MgCl_2$ and 1 mM ATP and washed with HBS. AMPK was eluted with 0.15 mg/ml 3×FLAG peptide and dialysed against HBS containing 10 % (v/v) glycerol and 1 mM DTT.

Competitive elution of AMPKγ2 from ATP-Sepharose. GST-AMPKγ2 (247-569) was absorbed onto γ-phosphate linked ATP-Sepharose (a gift from Tim Haystead, Duke University (Davies, et al., 1994)) for 2 h at 4°C. After washing with HBS to remove unbound protein, resin containing immobilised GST-AMPKγ2 was dispensed into tubes and eluted with the indicated ligands in HBS (100 µl) for 1 h at 4°C with constant mixing. Resin was pelleted at low speed and the resulting supernatant analysed for the presence of AMPKγ2 by SDS-PAGE and staining with colloidal Coomassie (Generon).

Isolation of primary hepatocytes. Hepatocytes were isolated from fasted mice by collagenase digestion (Berry and Friend, 1969). Cells were seeded in M199 containing 100 U/ml penicillin G, 100 µg/ml streptomycin, 0.1 % (w/v) BSA, 10 % (v/v) FBS, 10 nM insulin, 200 nM triiodothyronine and 100 nM dexamethasone. Post attachment (3-4 h), cells were cultured overnight in M199 supplemented with antibiotics and 100 nM dexamethasone and used for experiments the following morning.

Western blotting. Cells were stimulated as indicated, rinsed with ice-cold PBS and lysed with 50 mM tris pH 7.5, 1 mM EDTA, 1 mM EGTA, 0.27 M sucrose, 1 % (w/v) Triton X-100, 20 mM glycerol-2-phosphate, 50 mM NaF, 5 mM $Na_4P_2O_7$, 1 mM Na_3VO_4, 1 mM DTT, 0.5 mM PMSF, 1 mM benzamidine and 1 µM microcystin-LR. Lysates were clarified at 3000 g for 5 min at 4ºC and normalised using Bradford reagent and BSA as standard. Lysates (20 µg) were denatured in Laemlli buffer, separated on 7.5 % tris-glycine gels and transferred to PVDF in Towbin buffer. Membranes were blocked in 5 % (w/v) milk for 1 h at RT and incubated overnight in the indicated primary antibodies at 4ºC. Membranes were developed using HRP-conjugated antibodies and ECL reagent.

Nucleotide measurements. Cultured hepatocytes were incubated with the indicated compounds and washed with ice-cold 0.9 % (w/v) saline before extraction in 5 % perchloric acid. Precipitated material was removed by centrifugation (13,000 RPM, 3 min) and supernatant neutralised with 1:1 trioctylamine:Freon-113 (Khym, 1975). Nucleotides were separated and quantified by capillary electrophoresis using a Beckman P/ACE instrument as previously described (Hawley, et al., 2010).

Additional enzyme assays. Glycogen phosphorylase (EC 2.4.1.1) was assayed in the physiological direction in reactions containing 20 mM BES pH 6.8, 20 mM phosphate, 1 mM $NADP^+$, 0.5 mM $MgCl_2$, 0.1 % glycogen, 4 µM glucose-1,6-bisphosphate, 0.5 mM DTT, 0.025 % BSA, 0.6 U/ml phosphoglucomutase and 3 U/ml glucose-6-phosphate dehydrogenase (G6PDH). FBP1 (EC 3.1.3.11) was assayed in reactions containing 50 mM TES pH 7.4, 0.1 M KCl, 0.05 mM EDTA, 2 mM $(NH_4)_2SO_4$, 0.15 mM $NADP^+$, 2 mM $MgCl_2$, 0.04 % BSA, 2 mM 2-mercaptoethanol, 25 µM fructose-1,6-bisphosphate, 0.8 U/ml phosphoglucoisomerase and 0.5 U/ml G6PDH. PFK1 (EC 2.7.1.11) was assayed in reactions containing 50 mM TES pH 7.4, 2 mM $MgCl_2$, 0.05 % BSA, 2 mM 2-mercaptoethanol, 0.5 mM fructose-6-phosphate, 1 mM ATP, 0.2 mM NADH, 0.5 U/ml aldolase, 15 U/ml triosephosphate isomerase and 2 U/ml α-glycerophosphate dehydrogenase. AMPD1 (EC 3.5.4.6) was assayed in reactions containing 50 mM imidazole pH 7, 0.15 mM KCl, 1 mM DTT, 0.05 % BSA, 1 mM ATP, 5 mM 2-oxoglutarate, 0.2 mM NADH, 0.5 mM AMP and 5 U/ml glutamate dehydrogenase. AK (EC 2.7.4.3) was assayed in the direction of ADP formation in reactions containing 50 mM TES pH 7.4, 100 mM KCl, 10 mM $MgCl_2$, 0.05 % BSA, 2 mM 2-mercaptoethanol, 1 mM phosphoenolpyruvate, 0.5 mM AMP, 1 mM ATP, 0.2 mM NADH, 3 U/ml pyruvate kinase and 3 U/ml lactate dehydrogenase. All assays were performed in a final volume of 250 µl and were monitored by changes in fluorescence (FBP1 - λ_{ex} 340nm, λ_{em} 450nm) or A_{340}. 5'-Nucleotidase (EC 3.1.3.5) was assayed in reactions containing 25 mM TES pH 7.4, 5 mM $MgCl_2$ and 100 µM AMP. Liberated phosphate was determined using malachite green reagent. Acetate CoA ligase (EC 6.2.1.1) was assayed in reactions containing 50 mM TES pH 7.4, 0.1 mM EDTA, 5 mM $MgCl_2$, 4 mM ATP, 0.2 mM coenzyme A and 5 mM DTT. The formation of pyrophosphate (PP_i) was monitored by reduction of PP_i-molybdate complexes using 2-mercaptoethanol and Fiske-Subbarow reducer (Kuang, et al., 2007).

Kinase inhibition screening. Was performed by the International Centre for Kinase Profiling (Dundee) using [γ-^{33}P] phosphotransferase assays (MRC Protein phosphorylation and Ubiquitylation Unit, http://www.kinase-screen.mrc.ac.uk/).

Construction of polycistronic AMPK expression vectors

The coding regions of AMPKα1 (NM_006251), AMPKα2 (NM_006252), AMPKβ1 (NM_006253), and AMPKγ1 (NM_002733) were amplified from brain RNA (Agilent) using Superscript III RT-PCR kit (Invitrogen). The resulting PCR products were ligated into intermediate vectors using Strataclone PCR cloning kit (Agilent). Sequences were verified utilising the BigDyeR Terminator 3.1 kit on a 3500XL Genetic analyser (ABI-Invitrogen). Site-directed mutagenesis was carried out according to the Quikchange method (Stratagene) using KOD polymerase (Novagen).

Tricistronic expression vectors were constructed using a strategy inspired by Neumann *et al* (Neumann, et al., 2003). The multiple cloning site of pET15-b (Novagen) was modified using standard techniques to incorporate a *Not1* site into the polylinker. The open reading frame of AMPKα1 and AMPKα2 was inserted after the 6×His tag as a *Bamh1-Not1* fragment. AMPK β1 (containing 5' bacterial expression enhancer elements added in the 5' end oligo) was amplified by PCR and inserted in the *Not1* site as an *Eag1-Not1* fragment, (destroying one

side of the *Not1* site). Finally, AMPKγ1 (containing 5' bacterial enhancer elements in the 5' end oligo) was amplified by PCR and inserted into the *Not1* site of the vectors already containing 6×His-α2 and β1 or 6×His-α1 and β1 as a *Not1-Not1* fragment (primer sequences available on request).

Construction of AMPKα subunit chimeras

AMPKα2/α1 CAT (α1 M1-F265 + α2 K255-end)

In order to switch amino acids 266-560 of AMPKα1 with amino acids 255-552 of AMPKα2 the *Pme-Not1* fragment of the tricistronic vectors were swapped. The coding region of AMPKγ1 was cloned after the AMPKβ1 region as a *Not1-Not1* fragment as described above.

AMPKα2/α1 REG (α2 M1-F254 + α1 K266-end)

The amino acids of AMPKα2 from 255-552 were replaced with the amino acids of AMPKα1 266-560 with the same cloning strategy as described above.

AMPKα2/α1 HOOK (α2 M1-P378 + α1 R384-E391 + α2 L387-end)

The chimera containing the changes in AMPKα2 K379/R, C382/H, P383/T, A386/E were made by site-directed mutagenesis. Substituted amino acids are highlighted in red in the alignment (see below).

AMPKα2/α1 LOOP (α2 M1-P347 + α1 D359-G401 + α2 V397-end)

In order to create a chimera containing a 43 AA substitution in the AMPKα2 linker region, synthetic DNA was ordered from Invitrogen replacing the *Pme1-Not1* region as a *Pme-Not1* fragment. Then the coding regions of the AMPKβ1 and AMPKγ1 subunits were inserted using the same strategy as described above.

All constructs were verified by capillary sequencing.

Alignment of chimeric AMPK alpha sequences

```
α2/α1 CAT    1 MRRLSSWRKMATAEKQKHDGRVKIGHYILGDTLGVGTFGKVKVGKHELTGHKVAVKILNR
α2/α1 REG    1 -----------MAEKQKHDGRVKIGHYVLGDTLGVGTFGKVKIGEHQLTGHKVAVKILNR
α2/α1 HOOK   1 -----------MAEKQKHDGRVKIGHYVLGDTLGVGTFGKVKIGEHQLTGHKVAVKILNR
α2/α1 LOOP   1 -----------MAEKQKHDGRVKIGHYVLGDTLGVGTFGKVKIGEHQLTGHKVAVKILNR

α2/α1 CAT   61 QKIRSLDVVGKIRREIQNLKLFRHPHIIKLYQVISTPSDIFMVMEYVSGGELFDYICKNG
α2/α1 REG   50 QKIRSLDVVGKIKREIQNLKLFRHPHIIKLYQVISTPTDFFMVMEYVSGGELFDYICKHG
α2/α1 HOOK  50 QKIRSLDVVGKIKREIQNLKLFRHPHIIKLYQVISTPTDFFMVMEYVSGGELFDYICKHG
α2/α1 LOOP  50 QKIRSLDVVGKIKREIQNLKLFRHPHIIKLYQVISTPTDFFMVMEYVSGGELFDYICKHG

α2/α1 CAT  121 RLDEKESRRLFQQILSGVDYCHRHMVVHRDLKPENVLLDAHMNAKIADFGLSNMMSDGEF
α2/α1 REG  110 RVEEMEARRLFQQILSAVDYCHRHMVVHRDLKPENVLLDAHMNAKIADFGLSNMMSDGEF
α2/α1 HOOK 110 RVEEMEARRLFQQILSAVDYCHRHMVVHRDLKPENVLLDAHMNAKIADFGLSNMMSDGEF
α2/α1 LOOP 110 RVEEMEARRLFQQILSAVDYCHRHMVVHRDLKPENVLLDAHMNAKIADFGLSNMMSDGEF
```

```
α2/α1 CAT  181 LRTSCGSPNYAAPEVISGRLYAGPEVDIWSSGVILYALLCGTLPFDDDHVPTLFKKICDG
α2/α1 REG  170 LRTSCGSPNYAAPEVISGRLYAGPEVDIWSCGVILYALLCGTLPFDDEHVPTLFKKIRGG
α2/α1 HOOK 170 LRTSCGSPNYAAPEVISGRLYAGPEVDIWSCGVILYALLCGTLPFDDEHVPTLFKKIRGG
α2/α1 LOOP 170 LRTSCGSPNYAAPEVISGRLYAGPEVDIWSCGVILYALLCGTLPFDDEHVPTLFKKIRGG

α2/α1 CAT  241 IFYTPQYLNPSVISLLKHMLQVDPMKRATIKDIREHEWFKQDLPSYLFPEDPSYDANVID
α2/α1 REG  230 VFYIPEYLNRSVATLLMHMLQVDPLKRATIKDIREHEWFKQDLPKYLFPEDPSYSSTMID
α2/α1 HOOK 230 VFYIPEYLNRSVATLLMHMLQVDPLKRATIKDIREHEWFKQDLPSYLFPEDPSYDANVID
α2/α1 LOOP 230 VFYIPEYLNRSVATLLMHMLQVDPLKRATIKDIREHEWFKQDLPSYLFPEDPSYDANVID

α2/α1 CAT  301 DEAVKEVCEKFECTESEVMNSLYSGDPQDQLAVAYHLIIDNRRIMNQASEFYLASSPPSG
α2/α1 REG  290 DEALKEVCEKFECSEEEVLSCLYNRNHQDPLAVAYHLIIDNRRIMNEAKDFYLATSPP--
α2/α1 HOOK 290 DEAVKEVCEKFECTESEVMNSLYSGDPQDQLAVAYHLIIDNRRIMNQASEFYLASSPPSG
α2/α1 LOOP 290 DEAVKEVCEKFECTESEVMNSLYSGDPQDQLAVAYHLIIDNRRIMNQASEFYLASSPP--

α2/α1 CAT  361 SFMDDSAMHIPPGLKPHPERMPPLIADSPKARCPLDALNTTKPKSLAVKKAKWHLGIRSQ
α2/α1 REG  348 ----DSFLDDHHLTRPHPERVPFLVAETPRARHTLDELNPQKSKHQGVRKAKWHLGIRSQ
α2/α1 HOOK 350 SFMDDSAMHIPPGLKPHPERMPPLIADSPRARHTLDELNTTKPKSLAVKKAKWHLGIRSQ
α2/α1 LOOP 348 ----DSFLDDHHLTRPHPERVPFLVAETPRARHTLDELNPQKSKHQGVKKAKWHLGIRSQ

α2/α1 CAT  421 SKPYDIMAEVYRAMKQLDFEWKVVNAYHLRVRRKNPVTGNYVKMSLQLYLVDNRSYLLDF
α2/α1 REG  404 SRPNDIMAEVCRAIKQLDYEWKVVNPYYLRVRRKNPVTSTYSKMSLQLYQVDSRTYLLDF
α2/α1 HOOK 410 SKPYDIMAEVYRAMKQLDFEWKVVNAYHLRVRRKNPVTGNYVKMSLQLYLVDNRSYLLDF
α2/α1 LOOP 404 SKPYDIMAEVYRAMKQLDFEWKVVNAYHLRVRRKNPVTGNYVKMSLQLYLVDNRSYLLDF

α2/α1 CAT  481 KSIDDEVVEQRSGSSTPQRSCSAAGLHRPRSSFDSTTAESHSLSGSLTGSLTGSTLS--S
α2/α1 REG  464 RSIDDEITEAKSGTATPQRSGSVSNYRSCQRSDSDAEAQGKSSEVSLTSSVTSLDSSPVD
α2/α1 HOOK 470 KSIDDEVVEQRSGSSTPQRSCSAAGLHRPRSSFDSTTAESHSLSGSLTGSLTGSTLS--S
α2/α1 LOOP 464 KSIDDEVVEQRSGSSTPQRSCSAAGLHRPRSSFDSTTAESHSLSGSLTGSLTGSTLS--S

α2/α1 CAT  539 VSPRLGSHTMDFFEMCASLITTLAR
α2/α1 REG  524 LTPRPGSHTIEFFEMCANLIKILAQ
α2/α1 HOOK 528 VSPRLGSHTMDFFEMCASLITTLAR
α2/α1 LOOP 522 VSPRLGSHTMDFFEMCASLITTLAR
```

Supplemental References:

Berry, M.N., and Friend, D.S. (1969). High-yield preparation of isolated rat liver parenchymal cells: a biochemical and fine structural study. J Cell Biol 43, 506-520.

Davies, S.P., Hawley, S.A., Woods, A., Carling, D., Haystead, T.A., and Hardie, D.G. (1994). Purification of the AMP-activated protein kinase on ATP-gamma-sepharose and analysis of its subunit structure. European journal of biochemistry / FEBS 223, 351-357.

Gomez-Galeno, J.E., Dang, Q., Nguyen, T.H., Boyer, S.H., Grote, M.P., Sun, Z., Chen, M., Craigo, W.A., van Poelje, P.D., MacKenna, D.A., et al. (2010). A potent and selective AMPK activator that inhibits de novo lipogenesis. ACS Med. Chem. Lett. 1, 478-482.

Gouet, P., Robert, X., and Courcelle, E. (2003). ESPript/ENDscript: Extracting and rendering sequence and 3D information from atomic structures of proteins. Nucleic acids research 31, 3320-3323.

Hawley, S.A., Boudeau, J., Reid, J.L., Mustard, K.J., Udd, L., Makela, T.P., Alessi, D.R., and Hardie, D.G. (2003). Complexes between the LKB1 tumor suppressor, STRAD alpha/beta and MO25 alpha/beta are upstream kinases in the AMP-activated protein kinase cascade. Journal of biology 2, 28.

Hawley, S.A., Davison, M., Woods, A., Davies, S.P., Beri, R.K., Carling, D., and Hardie, D.G. (1996). Characterization of the AMP-activated protein kinase kinase from rat liver, and identification of threonine-172 as the major site at which it phosphorylates and activates AMP-activated protein kinase. J. Biol. Chem. 271, 27879-27887.

Hawley, S.A., Ross, F.A., Chevtzoff, C., Green, K.A., Evans, A., Fogarty, S., Towler, M.C., Brown, L.J., Ogunbayo, O.A., Evans, A.M., et al. (2010). Use of cells expressing gamma subunit variants to identify diverse mechanisms of AMPK activation. Cell Metab 11, 554-565.

Khym, J.X. (1975). An analytical system for rapid separation of tissue nucleotides at low pressures on conventional anion exchangers. Clin Chem 21, 1245-1252.

Kuang, Y., Salem, N., Wang, F., Schomisch, S.J., Chandramouli, V., and Lee, Z. (2007). A colorimetric assay method to measure acetyl-CoA synthetase activity: application to woodchuck model of hepatitis virus-induced hepatocellular carcinoma. J Biochem Biophys Methods 70, 649-655.

Needleman, S.B., and Wunsch, C.D. (1970). A general method applicable to the search for similarities in the amino acid sequence of two proteins. Journal of molecular biology 48, 443-453.

Neumann, D., Woods, A., Carling, D., Wallimann, T., and Schlattner, U. (2003). Mammalian AMP-activated protein kinase: functional, heterotrimeric complexes by co-expression of subunits in Escherichia coli. Protein Expr Purif 30, 230-237.

Sakamoto, K., Zarrinpashneh, E., Budas, G.R., Pouleur, A.C., Dutta, A., Prescott, A.R., Vanoverschelde, J.L., Ashworth, A., Jovanovic, A., Alessi, D.R., et al. (2006). Deficiency of

LKB1 in heart prevents ischemia-mediated activation of AMPKalpha2 but not AMPKalpha1. American journal of physiology. Endocrinology and metabolism 290, E780-788.

Smiley, K.L., Jr., Berry, A.J., and Suelter, C.H. (1967). An improved purification, crystallization, and some properties of rabbit muscle 5'-adenylic acid deaminase. J Biol Chem 242, 2502-2506.
